# The oral-gastric microbial axis in children: salivary signatures as non-invasive biomarkers for pediatric gastritis and *Helicobacter pylori* infection

**DOI:** 10.3389/fcimb.2026.1934250

**Published:** 2026-09-22

**Authors:** Ji Hyun Seo, Ji Sook Park, Jung Je Park, Young-Sool Hah

**Affiliations:** 1 Department of Pediatrics, Gyeongsang National University College of Medicine, Jinju, Republic of Korea; 2 Institute of Medical Science, Gyeongsang National University College of Medicine, Jinju, Republic of Korea; 3 Biomedical Research Institute, Gyeongsang National University Hospital, Jinju, Republic of Korea; 4 Department of Otorhinolaryngology-Head and Neck Surgery, Gyeongsang National University College of Medicine, Jinju, Republic of Korea

**Keywords:** oral-gastric axis, pediatric gastritis, *Helicobacter pylori*, salivary microbiome, non-invasive biomarkers, oral microbiome, dysbiosis

## Abstract

*Helicobacter pylori* is commonly acquired in childhood, with the mouth a presumed portal of entry, although the relative contributions of oral-oral, gastro-oral, and fecal-oral transmission remain unresolved. It is a principal cause of pediatric gastritis, yet most infected children are asymptomatic, and the pediatric mucosa mounts a tolerogenic response rather than the aggressive inflammation seen in adults. In the pathway defined by pediatric guidelines, diagnosis depends on endoscopy with multiple gastric biopsies, an invasive reference standard not suitable for serial assessment; non-invasive tests are reserved largely for confirming eradication and have age-specific limitations in young children. This narrative review, supported by a structured literature search, develops the oral-gastric microbial axis as an organizing framework for non-invasive pediatric assessment, on the premise—not yet demonstrated longitudinally—that early oral colonization shapes later gastric disease: the two compartments are ecologically continuous, and acid-tolerant oral bacteria that survive gastric transit provide a pro-inflammatory metabolic input to the mucosa. We evaluate candidate salivary and oral signatures—taxonomic, *H. pylori*-specific, and metabolic—and grade each on two axes: the population generating the evidence (pediatric, adult, or non-human) and the stage of biomarker development reached, each tied to a stated target condition. The picture is asymmetric: the connectivity argument is well supported and the mechanistic account coherent, but the model has not been demonstrated end-to-end in children. A few signatures have genuine pediatric support, and salivary assays targeting the *H. pylori* 23S rRNA gene reach the clinical-assay stage on pediatric evidence, with sensitivity of 87-94% but specificity ranging from 80% to 100% in the two cohorts with recoverable two-by-two data; no candidate has been independently validated in children, most evidence is adult-derived, and whether the oral cavity is a true *H. pylori* reservoir remains unresolved. Because a single, unreplicated comparative study indicates that *H. pylori* remodels the gastric microbiota differently in children than in adults, adult signatures cannot be assumed transferable. We conclude that oral-gastric signatures currently suit research stratification rather than clinical decision-making, and specify the longitudinal, multi-omics, viability-resolved studies needed to advance them toward clinical validity.

## 1 Introduction

*Helicobacter pylori* colonizes more than half of the world’s population, is acquired in childhood, with ingestion through the mouth as the presumed portal of entry and intrafamilial spread, especially from mother to child, as the most frequently reported transmission pattern (Elghannam et al., 2024), and is a principal cause of chronic gastritis and peptic ulcer disease in children (Hooi et al., 2017; Yuan et al., 2022; Lupu et al., 2025). It is also the dominant cause of gastric adenocarcinoma: classified by the International Agency for Research on Cancer as a Group 1 (definite) human carcinogen (IARC Working Group on the Evaluation of Carcinogenic Risks to Humans, 1994), it initiates the stepwise Correa cascade from chronic gastritis through atrophy, metaplasia, and dysplasia to cancer (Correa, 1992), and population-based randomized eradication trials now show that clearing the infection measurably lowers gastric-cancer incidence (Ford et al., 2020; Pan et al., 2024); the most recent global consensus accordingly endorses population-level *H. pylori* screening and eradication as a gastric-cancer prevention strategy (Liou et al., 2025). Because that cascade begins in childhood, the pediatric window is where interception would matter most. Yet the pediatric setting differs from the adult one in ways that shape everything downstream: most infected children are asymptomatic, and the dominant inflammatory response in the pediatric stomach is regulatory and tolerogenic rather than aggressive (Harris et al., 2008; Brawner et al., 2017). The clinical task is therefore not simply to detect the organism but to identify, non-invasively and without overtreating, the minority of children in whom infection is driving disease.

Current practice is poorly suited to that task. The joint European Society for Pediatric Gastroenterology, Hepatology, and Nutrition and North American Society for Pediatric Gastroenterology, Hepatology, and Nutrition (ESPGHAN/NASPGHAN) guidelines make the diagnosis of *H. pylori* deliberately rigorous and invasive—it must be biopsy-based, requiring at least six gastric biopsies obtained at upper endoscopy—and they explicitly recommend against a test-and-treat strategy in children, because the goal is to establish the cause of a child’s symptoms rather than merely to document the bacterium (Homan et al., 2024). Within this guideline-defined pathway, endoscopy stands as the diagnostic gatekeeper: definitive, but invasive, costly, and impractical for the repeated sampling that monitoring would demand. Statements about endoscopic dependence throughout this review should be read as applying to that pathway; in epidemiological surveys, in screening programs, and in settings where these guidelines are not applied, non-invasive testing predominates.

The non-invasive alternatives that exist do not close the gap. Guidelines advise against non-invasive testing for initial diagnosis and reserve the 13C-urea breath test (UBT) and the monoclonal stool antigen test mainly for confirming eradication (Homan et al., 2024). The same guidelines recommend against antibody-based (serological) tests in the clinical setting, because circulating anti-*H. pylori* antibodies can persist for months to years after the infection has cleared, and therefore cannot distinguish between active and past infection (Homan et al., 2024). The UBT carries an age-specific weakness directly relevant here: in children younger than six years, it tends to yield high false-positive rates and must be interpreted cautiously (Kindermann et al., 2000). Strikingly, one mechanism of UBT failure is itself a manifestation of the oral-gastric relationship this review concerns—urease-active oral bacteria that have colonized the stomach can generate persistently false-positive UBT results even when every *H. pylori*-specific test is negative (Nishino et al., 2023). Where the non-invasive tests are used, their pediatric performance is good but imperfect: pooled stool-antigen sensitivity and specificity are about 92% and 94% in children (Zhou et al., 2014), and head-to-head pediatric studies put the 13C-UBT and stool-antigen test on a par with biopsy for active infection (Kato et al., 2004). Yet in real-world multicenter pediatric cohorts, the specificity of both tests can fall sharply, to roughly 55–67% against histology (Raso et al., 2026)—a gap precisely the kind a more biologically grounded signature might close. The very confounder that undermines an existing non-invasive test points toward the microbial axis that a better test might exploit.

Two further developments widen the relevant question beyond *H. pylori* alone. First, a substantial fraction of cases of chronic gastritis are *H. pylori*-negative, suggesting that other microbial factors contribute to gastric inflammation (Cui et al., 2019). Second, the gastric microbiota is now understood to be ecologically continuous with the oral cavity: the genera most consistently found in the stomach—*Fusobacterium*, *Streptococcus*, *Haemophilus*, *Neisseria*, *Prevotella*, and *Veillonella*—are overwhelmingly oral-associated, and the stomach functions as a checkpoint through which oral microbes transit (Xia et al., 2025). This oral-gastric microbial axis is the organizing concept of the present review: if gastric microbial status is partly reflected in, and continuously seeded by, the oral cavity, then saliva and other oral specimens—trivially accessible in children—become candidate windows onto gastric disease. The overall framework of this review is summarized in **Figure 1**.

**Figure 1:**
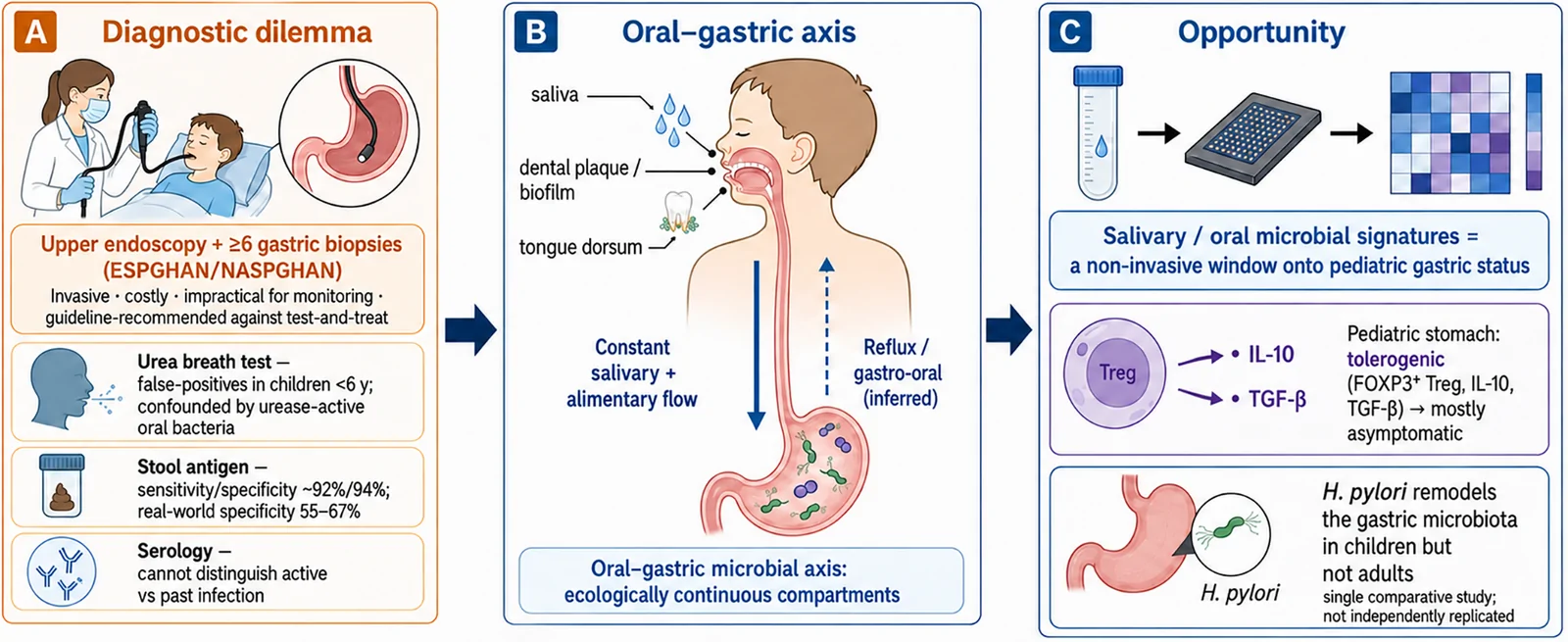
Overview of the oral-gastric microbial axis in children. **(A)** The diagnostic dilemma: endoscopy with ≥6 biopsies is the invasive gold standard, while the non-invasive alternatives (urea breath test, stool antigen, serology) carry the limitations indicated. **(B)** The oral-gastric axis: the oral cavity and stomach are ecologically continuous compartments; the solid arrow denotes the constant salivary and alimentary flow that seeds the stomach, and the dashed arrow denotes inferred reflux/gastro-oral transit. **(C)** The opportunity: salivary and oral microbial signatures, read by multi-omics platforms, are proposed as a non-invasive window into pediatric gastric status, which is shaped by a tolerogenic immune phenotype and by remodeling of the gastric microbiota associated with *H. pylori* that differs from the adult pattern, an observation derived from a single comparative study that has not been independently replicated. ESPGHAN, European Society for Pediatric Gastroenterology, Hepatology and Nutrition; NASPGHAN, North American Society for Pediatric Gastroenterology, Hepatology and Nutrition; FOXP3, forkhead box P3; IL-10, interleukin-10; TGF-β, transforming growth factor β.

The promise of that idea is undercut by a specific and consequential gap in the literature. The relevant evidence is overwhelmingly adult-derived, focused on gastric cancer or eradication outcomes, and based on invasive gastric specimens; the few studies that examine children remain cross-sectional and unvalidated. No oral or salivary microbial biomarker has been validated for pediatric gastritis in an independent cohort. Crucially, this is not merely a matter of fewer pediatric studies: in the only study to date that has directly compared infected children and adults, *H. pylori* infection was associated with significant remodeling of the gastric microbiota in children but not in adults (Brawner et al., 2017), so adult signatures cannot simply be scaled down—they may be qualitatively wrong when applied to children. This pediatric–adult divergence has not yet been replicated in an independent study with a matched adult comparison arm, and the supporting immune sub-analysis was small (n = 11 infected children). A review that foregrounds the pediatric, non-invasive, biomarker-oriented intersection—rather than the adult, gastric-cancer, tissue-based one that dominates the field—therefore addresses a genuine and unoccupied position. **Table 1** positions this review against recent reviews of the same axis, making explicit how its pediatric, gastritis-focused, saliva-based, and evidence-graded orientation differs from the adult, gastric-cancer, and tissue-based emphasis of prior work. In particular, it is distinct from the most closely related review in this journal—an adult-oriented, mechanistic account of the oral-gut axis and *H. pylori* (Chen et al., 2022b)—in being biomarker- and gastritis-oriented, pediatric, and explicitly evidence-graded. In contrast to prior reviews, which are predominantly adult-focused, gastric-cancer- or mechanism-oriented, and tissue-based, the present review is pediatric, gastritis-focused, saliva- or oral-specimen-based, and grades each candidate by evidence maturity.

**Table 1:** Positioning of this review relative to recent reviews of the oral–gastric/oral–gut microbial axis and gastric disease.

Review/reference	Age focus	Specimen type	Disease endpoint	Evidence grading	Diagnostic orientation
(Lupu et al., 2025)	Pediatric	Digestive-tract (multi-site)	*H. pylori* colonization/microbiome composition	No	Descriptive; not biomarker-oriented
(Xia et al., 2025)	Adult (not age-stratified)	Oral and gastric	Oral-gastric axis/general gastric health	No	Mechanistic/conceptual
(Chen et al., 2022b)	Adult	Oral and gut	*H. pylori* interactions across oral-gut axis	No	Mechanistic
(Xia et al., 2024)	Adult	Tongue coating, saliva, plaque (tissue/oral)	Gastric cancer	No	Cancer-risk biomarker discovery
This review	Pediatric	Saliva/oral (non-invasive)	Pediatric gastritis and *H. pylori* infection	Yes (explicit maturity grading, **Table 3**)	Non-invasive triage/stratification

Section 2 states how the literature was identified, appraised, and graded. This review is then organized to develop that position systematically. Section 3 examines how the oral and gastric communities are assembled and connected in childhood and confronts the unresolved controversy over whether the oral cavity is a true *H. pylori* reservoir. Section 4 sets out a proposed mechanistic model linking oral dysbiosis to pediatric gastric inflammation, including the distinctive tolerogenic immunology of the pediatric mucosa. Section 5, the analytical core, evaluates candidate salivary and oral microbial signatures—taxonomic, *H. pylori*-specific, and metabolic—together with the platforms that read them. Section 6 addresses translational realities: clinical fit, pediatric-specific confounders, and an explicit evidence-maturity grading of every candidate. Section 7 integrates these threads into a framework and a research roadmap. Throughout, the review distinguishes what is established in children from what is extrapolated from adults, and treats the field’s gaps and disputes as objects of analysis rather than obstacles to be glossed over.

## 2 Literature search and evidence appraisal

This is a narrative review supported by a structured search; it was not registered as a systematic review, and no quantitative synthesis was attempted. PubMed was searched to 11 June 2026. Searches were run separately for each subsection and, within each, separately for reviews and for primary studies, combining controlled vocabulary with free-text terms for *Helicobacter pylori* and gastric microbiota, the oral cavity and oral microbiome, and childhood, with a further block covering diagnostic accuracy, sequencing, metabolomics, volatile compounds, and machine learning for the biomarker subsections. Publication-date limits were set per subsection; all search strings and limits are given in **Supplementary Table S1**. No language restriction was applied. Reference lists of included articles, of the pediatric guidelines, and of the reviews compared in **Table 1** were hand-searched, which is how most pre-2010 sources were identified. The biomarker concept block did not include exosome or extracellular-vesicle terms, and one eligible pediatric study using that platform was identified by hand-searching during revision; **Supplementary Table S1** therefore records the searches actually run rather than an exhaustive strategy.

Eligible records were peer-reviewed primary studies, systematic reviews, or clinical practice guidelines addressing the oral or gastric microbiota, detection of *H. pylori* at an oral site, or a candidate non-invasive marker of gastric infection, gastritis, or gastric neoplasia. Adult, animal, and *in-vitro* studies were eligible only as mechanistic or comparative context and are labeled as such wherever they are cited; pediatric filters were deliberately omitted from the mechanism and biomarker searches so that the adult provenance of that evidence would remain visible rather than be concealed by the filter. Because a review that grades candidates is vulnerable to positive-outcome bias, the reservoir searches deliberately included terms that surface counter-evidence—persistence, recurrence, reinfection, coccoid forms, and the viable-but-non-culturable state—and discordant studies are retained in the text and tables rather than omitted; Section 3.3 sets the evidence for and against an oral reservoir side by side. Two authors screened and graded independently and resolved disagreements by returning to the primary articles.

Candidates are graded on two independent axes rather than under a single label, because the population that generated the evidence and the stage of development that evidence represents are separate questions. The first axis is the evidence population: pediatric, meaning at least one study in participants under 18 years reporting the marker for the stated target condition; adult; or non-human, which covers animal, cell-culture, isolate-level, and purely analytical work. The second is the stage of biomarker development, after Pepe et al. (2001): Phase 1, exploratory, with no standardized assay; Phase 2, a standardized assay applied to clinically obtained specimens with accuracy estimated against a reference standard; Phase 3, retrospective longitudinal measurement in specimens collected before the target condition was ascertained; Phase 4, prospective application in the intended-use population with a prespecified threshold; and Phase 5, a demonstrated effect on clinical decisions or outcomes. For readability, the four strata used in **Figure 2** map onto these phases as follows: mechanistic or analytical work and discovery-stage studies both fall within Phase 1, distinguished by whether the marker was measured in the non-invasive specimen intended for the eventual test; and clinical-assay studies reporting accuracy against a reference standard correspond to Phase 2. Independent-cohort validation is a strength within Phase 2 rather than a phase of its own; progression to Phases 3 to 5 is defined by design—prediagnostic longitudinal measurement, prospective application in the intended-use population, and demonstrated effect on outcomes—not by cohort independence.

**Figure 2:**
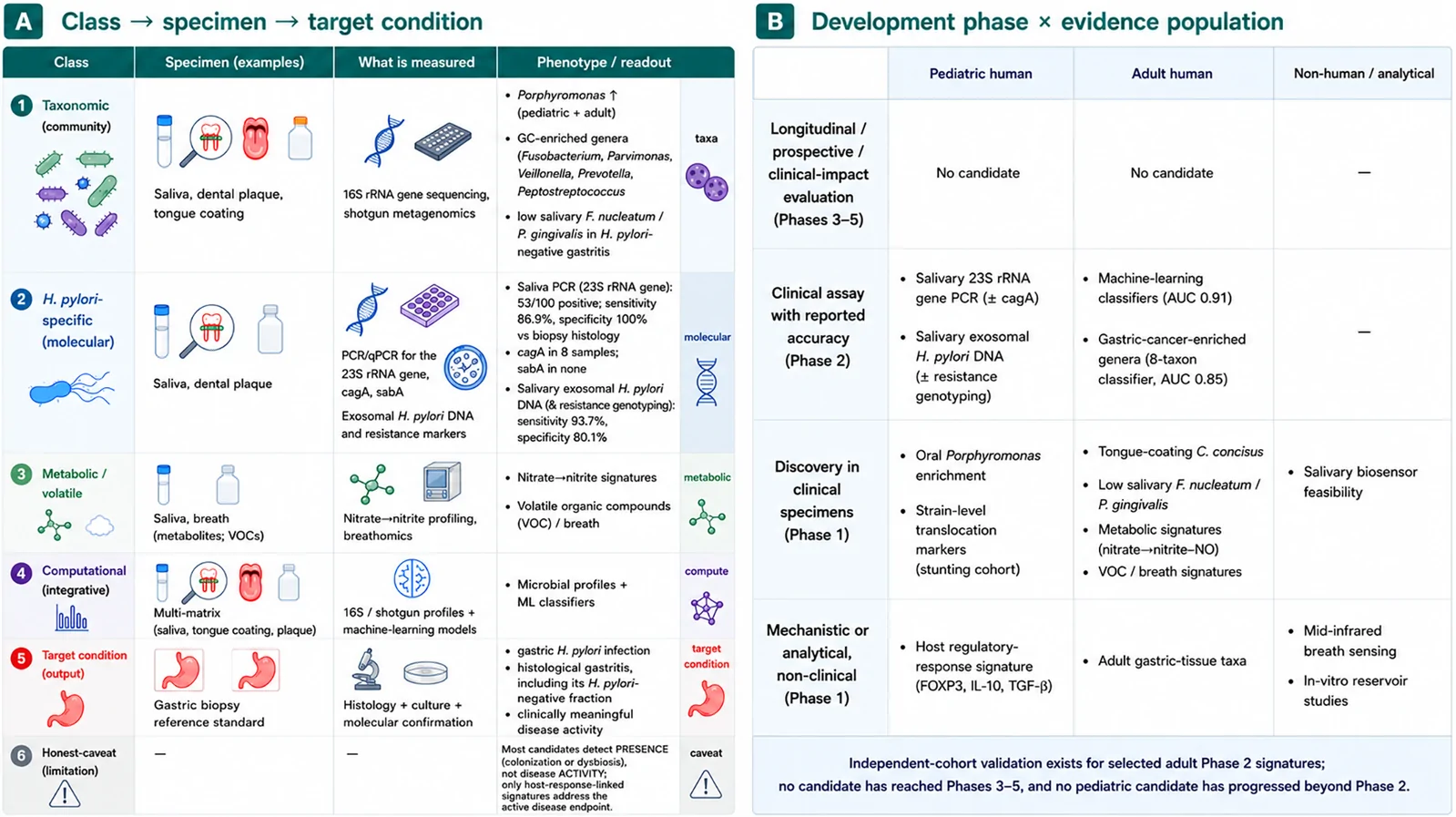
Specimen-analyte-target mapping and evidence status of candidate signatures. **(A)** Four candidate signature classes—taxonomic (community), *H. pylori*-specific molecular, metabolic/volatile, and computational/integrative—are traced from the specimens in which each is actually measured, through what is measured, to the readout, and each class lists only the specimens used for that analyte: 16S rRNA gene sequencing and shotgun metagenomics against saliva, dental plaque and tongue coating; nucleic-acid detection of the *H. pylori* 23S rRNA gene, *cagA* and *sabA*, and of exosomal *H. pylori* DNA and clarithromycin-resistance markers, against saliva and dental plaque; nitrate-nitrite profiling and breathomics against saliva and breath; and machine-learning models against multi-matrix input. Salivary nucleic-acid targets are not breath-based, so breath appears only against volatile and urea-derived analytes. Reported pediatric accuracy is given where available. The output row carries the three target conditions defined in Section 2, gastric *H. pylori* infection, histological gastritis including its *H. pylori*-negative fraction, and clinically meaningful disease activity, each of which must be prespecified together with its reference standard. The final row is a deliberate caveat: most candidates detect microbial presence (colonization/dysbiosis) rather than disease activity, and only host-response-linked signatures address the activity-level endpoint. **(B)** The same candidates are placed on the two grading axes of Section 2, with the evidence population across the columns (pediatric human, adult human, and non-human or analytical) and the stage of development down the rows. Each row label names the study design and the corresponding phase, so the panel can be read against **Tables 2** and **3** without cross-reference. Two pediatric candidates occupy the Phase 2 row, salivary 23S rRNA gene PCR and the salivary exosomal assay, and both address gastric infection rather than gastritis. The top row, covering Phases 3 to 5, is empty in every population: independent-cohort validation has been reported for selected adult gastric-cancer signatures and is recorded within Phase 2, but no candidate in any population has reached the longitudinal, prospective intended-use, or clinical-impact stages, and no pediatric candidate has progressed beyond Phase 2 at all, which is the review’s central gap. Taxa and gene symbols are italicized throughout; protein products are set in roman. Panel **(B)** carries no tier colors and no direction marks; the evidence population and the development phase are given by the row and column position. GC, gastric cancer; VOC, volatile organic compound; ML, machine learning.

Four rules govern application. The axes are always reported together and never collapsed into one term. A candidate supported only by adult data cannot be assigned a pediatric phase. A single cohort without internal validation does not advance beyond Phase 2, however large the reported effect. A candidate generated in gastric-cancer or premalignant cohorts is graded against that target condition, and its transfer to children is treated as an untested extrapolation. Where a primary study reports no accuracy metric, the phase is capped at Phase 1.

Finally, three target conditions are kept distinct throughout, because they are neither biologically nor diagnostically interchangeable and are routinely run together in this literature. Gastric *H. pylori* infection is the presence of the organism in the gastric mucosa, ascertained against a biopsy-based reference standard. Histological gastritis is mucosal inflammation graded on gastric biopsies, whether or not *H. pylori* is present; the *H. pylori*-negative fraction of pediatric chronic gastritis makes this a separate endpoint. Clinically meaningful disease activity is the state that would alter management. Every candidate in **Table 2** and **3** is tied to the target condition against which it was evaluated and to the reference standard used; reported accuracy is given where the primary study provides it and marked NR where it does not, and estimates we derived from counts reported in a primary study are identified as such.

**Table 2:** Candidate oral and salivary microbial taxa as non-invasive signatures, with the target condition, reference standard, and reported diagnostic accuracy for each.

Taxon	Direction	Target condition (reference standard)	Evidence population (design, n)	Specimen; platform	Reported diagnostic accuracy	Grade (population, phase)	Reference
Pediatric evidence							
*Porphyromonas*	Increased	Oral *H. pylori* carriage (oral PCR; no gastric reference standard)	Pediatric and adult; cross-sectional; 21 children	Saliva, dental plaque; 16S rRNA gene sequencing	NR (community comparison only)	Pediatric, Phase 1	(Ogaya et al., 2024)
Adult-derived evidence; not evaluated in children							
*Campylobacter concisus*	Increased	Gastritis and precancerous cascade (endoscopy with histopathology)	Adult; 78 gastritis and 50 controls, plus 38-patient detection cohort	Tongue coating, gastric fluid; shotgun metagenomics, qPCR	NR	Adult, Phase 1	(Cui et al., 2019); cross-reaction caution (Scholz et al., 2025)
*Fusobacterium* (*F. nucleatum*)	Increased in gastric cancer; decreased in *H. pylori*-negative gastritis saliva	Gastric carcinogenesis; *H. pylori*-negative chronic gastritis (gastric histopathology)	Adult; n per taxon NR	Gastric mucosa, saliva; 16S rRNA gene sequencing, qPCR	NR	Adult, Phase 1	(Coker et al., 2018; Contaldo et al., 2021)
*Prevotella*	Increased	Gastric cancer; *H. pylori*-negative gastritis (gastric histopathology)	Adult; n per taxon NR	Gastric mucosa, saliva; 16S rRNA gene sequencing	NR	Adult, Phase 1	(Ferreira et al., 2018; Hsieh et al., 2018; Zhang et al., 2023)
*Veillonella*	Increased	Gastric cancer and precancerous cascade (gastric histopathology)	Adult; n per taxon NR	Gastric mucosa; 16S rRNA gene sequencing	NR	Adult, Phase 1	(Castaño-Rodríguez et al., 2017; Chattopadhyay et al., 2023)
*Streptococcus*	Increased	Gastric cancer; gastritis (gastric histopathology)	Adult; n per taxon NR	Gastric mucosa, saliva; 16S rRNA gene sequencing	NR	Adult, Phase 1	(Zhang et al., 2023; Yang et al., 2025)
*Parvimonas*	Increased	Gastric carcinogenesis (gastric histopathology)	Adult; n per taxon NR	Gastric mucosa; 16S rRNA gene sequencing	NR	Adult, Phase 1	(Coker et al., 2018)
*Peptostreptococcus*	Increased	Gastric carcinogenesis (gastric histopathology)	Adult; n per taxon NR	Gastric mucosa; 16S rRNA gene sequencing	NR	Adult, Phase 1	(Ferreira et al., 2018)
*Neisseria*	Increased	*H. pylori*-negative gastritis (gastric histopathology)	Adult; n per taxon NR	Gastric mucosa; 16S rRNA gene sequencing	NR	Adult, Phase 1	(Hsieh et al., 2018; Zhang et al., 2023)

Direction indicates the reported change in the diseased state. Each candidate is tied to the target condition against which it was actually evaluated and to the reference standard used; NR indicates that the primary study did not report sensitivity, specificity, or an area under the curve for that target condition and population. Grades follow the two axes defined in Section 2. With the single exception of *Porphyromonas*, every taxon here is supported only by adult data, which is the review’s central gap statement; adult-derived entries are grouped separately for that reason and are not directly comparable with the pediatric entry. Several taxa change direction with disease context—*Fusobacterium*, for instance, is enriched in gastric cancer yet reduced in *H. pylori*-negative gastritis saliva, which is precisely why no single taxon can serve as a stand-alone classifier. No head-to-head comparison of these taxa in a common population has been reported, so no candidate is ranked against another. GC, gastric cancer; NR, not reported. A meta-analytic eight-taxon mucosal classifier incorporating several of these genera reached an area under the curve of 0.85 for gastric cancer versus superficial gastritis, but accuracy has not been reported for any single genus.

**Table 3:** Two-axis grading of candidate oral and salivary biomarkers: evidence population and stage of biomarker development, by target condition.

Candidate signature	Target condition	Evidence population	Development phase	Reported diagnostic accuracy	Principal limitation	Reference
Salivary exosomal *H. pylori* DNA by qPCR, with clarithromycin-resistance genotyping	Gastric *H. pylori* infection; clarithromycin resistance	Pediatric; n = 500, two centers	Phase 2	Sensitivity 93.7% (95% CI 88.7-96.9); specificity 80.1% (95% CI 75.5-84.2); PPV 68.5%; NPV 96.5%; confidence intervals calculated by us from the reported counts	No external validation; 68 false positives among 342 uninfected children, so a positive result is not confirmatory and the PPV is 68.5% at the observed prevalence; resistance genotyping agreed with gastric tissue in 103 of 113 (kappa 0.72)	(Song et al., 2026)
Salivary *H. pylori* 23S rRNA gene PCR, with or without *cagA*	Gastric *H. pylori* infection	Pediatric; n = 100, single center	Phase 2	Sensitivity 86.9% (95% CI 75.8-94.2); specificity 100% (95% CI 91.0-100); calculated by us from the reported counts	Single small cohort; reference standard was Giemsa histology alone; cannot distinguish oral residence from reflux-derived contamination; *cagA* detected in eight samples	(Samani et al., 2026)
Oral *Porphyromonas* enrichment	Oral *H. pylori* carriage	Pediatric and adult; 21 children	Phase 1	NR (community comparison only)	Cross-sectional; genus level only; no standardized assay or threshold; link to gastritis unproven	(Ogaya et al., 2024)
Tongue-coating *Campylobacter concisus*	Gastritis and precancerous cascade	Adult only	Phase 1	NR	No pediatric data; PCR cross-reaction with other *Campylobacter* species	(Cui et al., 2019; Scholz et al., 2025)
Low salivary *F. nucleatum* and *P. gingivalis*	*H. pylori*-negative chronic gastritis	Adult only; exploratory, n = 21	Phase 1	NR	Small exploratory cohort; direction opposite to that reported in gastric cancer; no pediatric data	(Contaldo et al., 2021)
Gastric-cancer-enriched genera (*Fusobacterium*, *Parvimonas*, *Veillonella*, *Prevotella*, *Peptostreptococcus*)	Gastric carcinogenesis, not pediatric gastritis	Adult only	Phase 2	Eight-taxon mucosal classifier, area under the curve 0.85 (gastric cancer versus superficial gastritis); not reported for any single genus	Reaches Phase 2 for a target condition and population other than those this review addresses; direction is context-dependent; transfer to children untested	(Liu et al., 2022); (Coker et al., 2018; Ferreira et al., 2018)
Metabolic signatures (enterosalivary nitrate-nitrite-nitric oxide pathway)	Mechanism; no clinical target condition defined in children	Adult and non-human	Phase 1	NR	Strongly diet-dependent; no pediatric gastritis-specific evaluation	(Hyde et al., 2014; Yang et al., 2025)
Volatile organic compound and breath signatures	Gastric *H. pylori* infection in adults	Adult only	Phase 1	NR	Platform-development stage; no pediatric data	(Flores Rangel et al., 2025)
Strain-level translocation markers	Oral-to-gut strain transfer, not gastritis	Pediatric; stunting cohort, n = 44	Phase 1	NR	Different cohort and target condition; not a deployable assay	(Yersin et al., 2026)
Machine-learning classifiers	Gastric cancer or digestive-tract tumours	Adult only; single-center and multicenter	Phase 2	Area under the curve 0.91 for a multicenter tongue-coating metaproteomic model and for an eight-genus salivary panel	Adult population and cancer endpoint throughout; no classifier developed or validated in children was identified, so transfer to pediatric gastritis is untested	(Huang et al., 2021); (Yuan et al., 2023; Chen et al., 2024; Kim et al., 2026)
Host regulatory-response signature (FOXP3, IL-10, TGF-beta)	Clinically meaningful disease activity (proposed; hypothesis H3)	Pediatric	Phase 1	NR	Hypothesis only; no assay or threshold defined; the only candidate addressing the activity-level endpoint	(Harris et al., 2008; Brawner et al., 2017)

Candidates are graded on two independent axes, as defined in Section 2. Evidence population: pediatric, at least one study in participants under 18 years reporting the marker for the stated target condition; adult, evidence restricted to adults; non-human, animal, cell-culture, or isolate-level work only. Development phase, after Pepe et al. (2001): Phase 1, exploratory, with no standardized assay; Phase 2, standardized assay applied to clinically obtained specimens with accuracy estimated against a reference standard; Phase 3, retrospective longitudinal; Phase 4, prospective application in the intended-use population with a prespecified threshold; Phase 5, demonstrated effect on clinical decisions or outcomes. Where a primary study reports no accuracy metric the phase is capped at Phase 1. That no candidate reaches Phase 3 or beyond, and that no candidate has been evaluated in children against histological gastritis or disease activity as the target condition, are themselves findings. No machine-learning classifier for an oral or salivary signature that was developed or validated in children was identified. NR, not reported; CI, confidence interval.

## 3 The oral-gastric axis in children: establishment, connectivity, and the reservoir question

Before the mechanistic and diagnostic arguments of the later sections can stand, three foundational questions must be settled: how the oral and gastric communities are assembled in childhood, how they are physically and ecologically connected, and whether the oral cavity is a genuine reservoir for *H. pylori* or merely a transient waystation. The third question is actively contested, and this section treats the controversy as a feature to be mapped rather than a difficulty to be smoothed over.

### 3.1 Assembly of the oral and gastric microbiota in childhood

*H. pylori* is overwhelmingly acquired in childhood, with the mouth as the presumed portal of entry, which makes the developmental assembly of the oral and gastric communities the relevant baseline for everything that follows (Yuan et al., 2022; Ogaya et al., 2024). The starting point, however, is that direct longitudinal data on how oral and gastric communities co-develop across the canonical pediatric windows—mode of delivery, breastfeeding, weaning, and tooth eruption—are sparse; most of what is known is inferred from cross-sectional snapshots rather than from longitudinal tracking. Where longitudinal pediatric data do exist, they are instructive: tracking 90 children from birth to seven years showed an ordered ecological succession in which diversity and richness climb with dental development and postnatal exposures (Dzidic et al., 2018), and a first-year infant-mother cohort found that the introduction of new nutrient sources, rather than tooth eruption per se, drove the rise in community complexity (Sulyanto et al., 2019). Independent cohorts reinforce this picture of an ordered, diet-shaped succession: the infant oral microbiome develops through reproducible colonization and succession events governed chiefly by neonatal diet (Blum et al., 2022), salivary communities continue to shift in composition across at least the first five years of life before approaching an adult-like state (Yama et al., 2024), and parallel multi-niche sampling of mother-partner-infant triads shows the oral and gut communities assembling along distinct, parent-influenced trajectories (Simonyté Sjödin et al., 2025). These are oral-cavity studies, however, with no paired gastric sampling, so the co-development of the two communities remains the gap this review identifies.

What can be stated with confidence is that the pediatric gastric community is developmentally labile in a way the adult community is not. In the absence of *H. pylori*, the gastric microbiota of children and adults is broadly similar, differing only in low-abundance taxa (Brawner et al., 2017); the developmental difference becomes visible specifically under perturbation, where the still-maturing pediatric gut and gastric microbiota are more susceptible to alteration than their adult counterparts, including after eradication therapy (Sung et al., 2020; Lupu et al., 2025). The implication is that childhood is not simply a smaller-scale version of the adult state but a distinct, more plastic ecological phase—the appropriate frame for asking how early oral colonization might leave a durable gastric mark.

### 3.2 Oral-gastric continuity and the niches of oral *H. pylori*

The two compartments are ecologically continuous. A systematic reading of gastric microbiota studies finds that the most consistently detected gastric genera—*Fusobacterium*, *Streptococcus*, *Haemophilus*, *Neisseria*, *Prevotella*, and *Veillonella*—are the same genera that dominate the oral cavity (Bik et al., 2006; Andersson et al., 2008; Maldonado-Contreras et al., 2011), and the stomach can be understood as a checkpoint along the digestive tract through which oral microbes transit (Xia et al., 2025), and the two compartments are increasingly analyzed together as a single oral-gut axis in which *H. pylori* is an active participant (Chen et al., 2022b). This continuity is not merely compositional overlap; it is the structural premise that makes a salivary window onto gastric status conceivable at all.

Within the oral cavity, *H. pylori* and its candidate markers occupy specific niches, and these niches are not equivalent. In children, *H. pylori* is more readily detected in dental plaque than in saliva, plausibly because salivary flow dilutes bacterial levels (Lupu et al., 2025); yet saliva remains a productive specimen, with PCR targeting the 23S rRNA gene detecting *H. pylori* in 53% of a pediatric endoscopy cohort (Samani et al., 2026). The genus-level signal also localizes: *Porphyromonas* is enriched in the oral cavity of *H. pylori*-positive children and is notably the only genus enriched in both children and adults in this context (Ogaya et al., 2024). That oral carriage may be more than incidental has been probed at the strain level: genotyping and restriction-fragment analysis have been used to test whether plaque and gastric *H. pylori* are the same organism within individual dyspeptic patients (Moosavian et al., 2023), and in young children, *H. pylori* has been detected within deep carious lesions, implicating the oral cavity as a possible source of infection in early life (Sruthi et al., 2023). The practical consequence is that specimen choice—plaque, saliva, or tongue surface—is a design variable that materially affects detection, and any biomarker claim must specify it.

### 3.3 The reservoir question: a genuine and unresolved controversy

Whether the oral cavity is a true *H. pylori* reservoir—a site of stable colonization that can reseed the stomach and drive reinfection—is the central dispute of this field, and the evidence cuts both ways. The review’s position is that the question remains open and that the competing lines of evidence warrant explicit presentation. The candidate oral niches and the competing evidence on the reservoir question are summarized in **Figure 3**.

**Figure 3:**
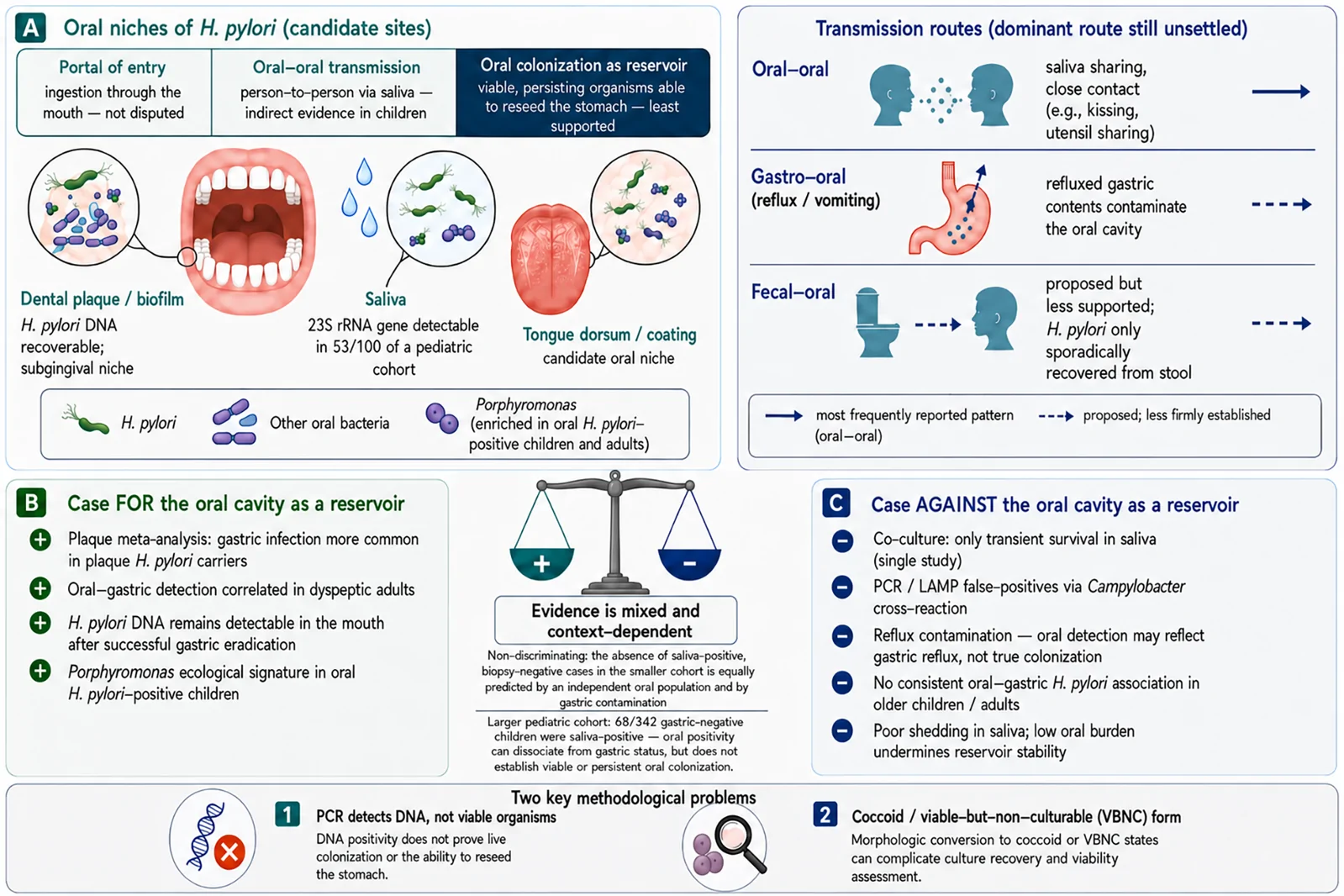
Oral niches of *H. pylori* and the reservoir controversy. **(A)** Candidate oral niches of *H. pylori*—dental plaque/biofilm, saliva, and the tongue dorsum—together with the oral genera relevant to its detection, including *Porphyromonas*. Three claims that this panel keeps distinct are the portal of entry (ingestion through the mouth), oral-oral transmission (person-to-person spread through saliva), and stable oral colonization as an extragastric reservoir; only the last requires viable, persisting organisms. Transmission routes: solid arrows denote the most frequently reported pattern (oral-oral), and dashed arrows denote proposed but less firmly established routes (gastro-oral and fecal-oral). **(B, C)** The competing evidence for **(B)** and against **(C)** the oral cavity as a stable *H. pylori* reservoir, weighed on a balance to convey that the evidence is mixed and context-dependent; plus and minus symbols mark supporting and opposing lines of evidence. Two notes beneath the balance record findings that fall on neither side. The absence of saliva-positive, biopsy-negative cases in the smaller pediatric cohort is equally predicted by an independent oral population and by gastric contamination; and in the larger pediatric cohort 68 of 342 gastric-negative children were saliva-positive, which shows that oral positivity can dissociate from gastric status without establishing viable or persistent oral colonization. The two recurring methodological problems that sustain the controversy are shown below; the first of these is that detection of *H. pylori* DNA establishes the presence of nucleic acid, not of viable or transmissible organisms. PCR, polymerase chain reaction; LAMP, loop-mediated isothermal amplification.

Three claims are routinely conflated in this literature and are kept separate here. Portal of entry refers to ingestion of *H. pylori* through the mouth; it is not seriously disputed, but it says nothing about whether the organism persists there. Oral-oral transmission refers to person-to-person spread through saliva; in children the supporting evidence is largely indirect, resting on intrafamilial clustering and strain sharing, and gastro-oral and fecal-oral routes remain plausible alternatives whose relative contribution has not been established. Oral colonization as an extragastric reservoir requires stable persistence of viable organisms at an oral site with the capacity to reseed the stomach; this is the strongest of the three claims and the least well supported. A fourth distinction is analytical rather than conceptual. Almost all oral studies rely on PCR, and detection of *H. pylori* DNA in saliva or dental plaque establishes the presence of nucleic acid, not of viable, culturable, or transmissible organisms. A positive oral result is therefore equally compatible with transient passage, with swallowed or refluxed gastric contents, and with amplification of DNA from non-viable cells, and the reservoir question cannot be settled by detection studies alone. The evidence reviewed below is grouped accordingly.

The case for a reservoir rests on detection and association. *H. pylori* DNA is recoverable from plaque, saliva, and the tongue dorsum across many studies; in children, salivary positivity is substantial and, in one cohort, no saliva-positive child was biopsy-negative, so oral detection was highly specific for gastric infection in that setting; a larger pediatric cohort using a two-test reference standard found the opposite, with 68 of 342 uninfected children salivary-positive, so the specificity of oral detection for gastric status is not settled and cannot be used to argue either for or against an oral reservoir (Samani et al., 2026). A meta-analysis of plaque studies found gastric infection to be significantly more common in people carrying *H. pylori* in dental plaque, framing plaque as a candidate extra-gastric reservoir from which the stomach could be re-seeded (Anand et al., 2025), and a cohort of dyspeptic adults showed a strong correlation between oral and gastric detection (Rasmussen et al., 2010). The most provocative observation is persistence after treatment: in one series *H. pylori* remained detectable in the oral cavity of nearly all patients despite successful gastric eradication, implying an oral compartment that systemic therapy does not clear (Czesnikiewicz-Guzik et al., 2005); in the same vein, a randomized trial tested whether adding nonsurgical periodontal therapy to standard eradication improves gastric clearance in patients carrying *H. pylori* within their subgingival biofilm (Behfarnia et al., 2025). The reproducible enrichment of *Porphyromonas* in *H. pylori*-positive children adds an ecological signature consistent with genuine oral residence rather than incidental contamination (Ogaya et al., 2024).

The case against is equally concrete and has been strengthened by recent work. Co-culture experiments in human saliva—the principal direct experimental evidence on this point to date, and from a single study—show that *H. pylori* can only transiently survive in the oral environment and may not be a permanent resident of the oral microbiota (Scholz et al., 2025)—a direct challenge to the stable-colonization premise. Notably, the same study also found that *H. pylori* survival in saliva was enhanced in co-culture with certain oral commensals, so this single *in-vitro* dataset is not unidirectional evidence against an oral niche (Scholz et al., 2025). The same line of work flags two confounders that inflate apparent oral positivity: non-invasive PCR and LAMP assays are prone to false positives through cross-reaction with *Campylobacter* species, and the oral cavity can be contaminated with gastric *H. pylori* via gastroesophageal reflux, so a positive oral result may reflect transient seeding from the stomach rather than an independent oral population (Scholz et al., 2025). Compounding this, some studies have failed to find a significant association between plaque *H. pylori* and gastric status within the same individual, underscoring the complexity of the relationship (Xia et al., 2025). Two further observations sharpen the case against a stable reservoir: when nested PCR detected *H. pylori* DNA in the mouths of 97% of patients, the distribution bore no relationship to gastric infection status, suggesting the organism may be a transient member of the normal oral microflora rather than a gastric-derived colonizer (Song et al., 2000); and others attribute oral detection to the handling of swallowed contaminants rather than true oral infection (Doré-Davin et al., 1999).

Two unresolved methodological problems sit beneath the controversy and explain why it persists. First is the culture-negative/PCR-positive discordance: molecular assays frequently detect *H. pylori* sequences in the absence of viable organisms, leaving it ambiguous whether the detected DNA represents living, colonizing bacteria or non-viable residues. Second is the organism’s capacity to adopt a coccoid form under aerobic conditions, which may persist in the environment (Scholz et al., 2025), raising the possibility of a viable-but-non-culturable state that standard culture would miss but may or may not be transmission-competent. Until detection methods can distinguish viable, colonizing *H. pylori* from transient or non-viable DNA, claims that the oral cavity is a definitive reservoir—or definitively not one—are premature. Recent metagenomic profiling that tracks oral and intragastric communities together through the peri-eradication period is beginning to characterize this relationship more directly (Matsumoto et al., 2026), but because the route by which the organism actually moves between hosts—with oral-oral spread (intrafamilial, especially mother-to-child) the most frequently reported pattern in children, and gastro-oral (reflux/vomiting) and fecal-oral routes proposed but less firmly established, since *H. pylori* is only sporadically recovered from stool—is itself unsettled (Duan et al., 2023), such data tighten rather than resolve the question.

For the purposes of this review, the reservoir controversy is not a side issue but a direct constraint on biomarker interpretation. If oral *H. pylori* is often transient or reflux-derived, then an oral *H. pylori*-specific marker may report gastric infection indirectly rather than indicating an independent oral focus—a distinction that changes how a positive test should be acted on. The broader oral-dysbiosis signatures developed in Section 5 are, for this reason, on firmer conceptual ground than oral *H. pylori* detection alone, because they do not depend on resolving whether the organism truly colonizes the mouth.

## 4 A proposed mechanistic model linking oral dysbiosis to pediatric gastric inflammation

The biomarker logic developed in Section 5 holds only if there is a mechanistic chain linking oral microbial changes to gastric disease in children; otherwise, a salivary signature is at best a correlate and at worst an artifact of shared sampling. This section sets out a proposed model of that chain in three steps: how oral bacteria physically reach and persist in the stomach, what they do metabolically once there, and how the pediatric mucosal immune response converts colonization into a disease phenotype that differs from the adult case. The proposed model is depicted in **Figure 4**. It is assembled from heterogeneous sources—pediatric human, adult human, animal, gastric-cancer, and *in-vitro* studies—and no study has demonstrated the complete sequence in children with histologically confirmed gastritis; the individual links differ substantially in how well they are established, and the evidence source for each is indicated in **Figure 4**. The model is therefore offered as a framework for testing rather than as a demonstrated causal sequence, and the three hypotheses in Section 7 are constructed to put its weakest links at risk. Throughout, the emphasis is on the proposed sequence rather than on a catalog, and on the points where pediatric evidence diverges from the adult literature.

**Figure 4:**
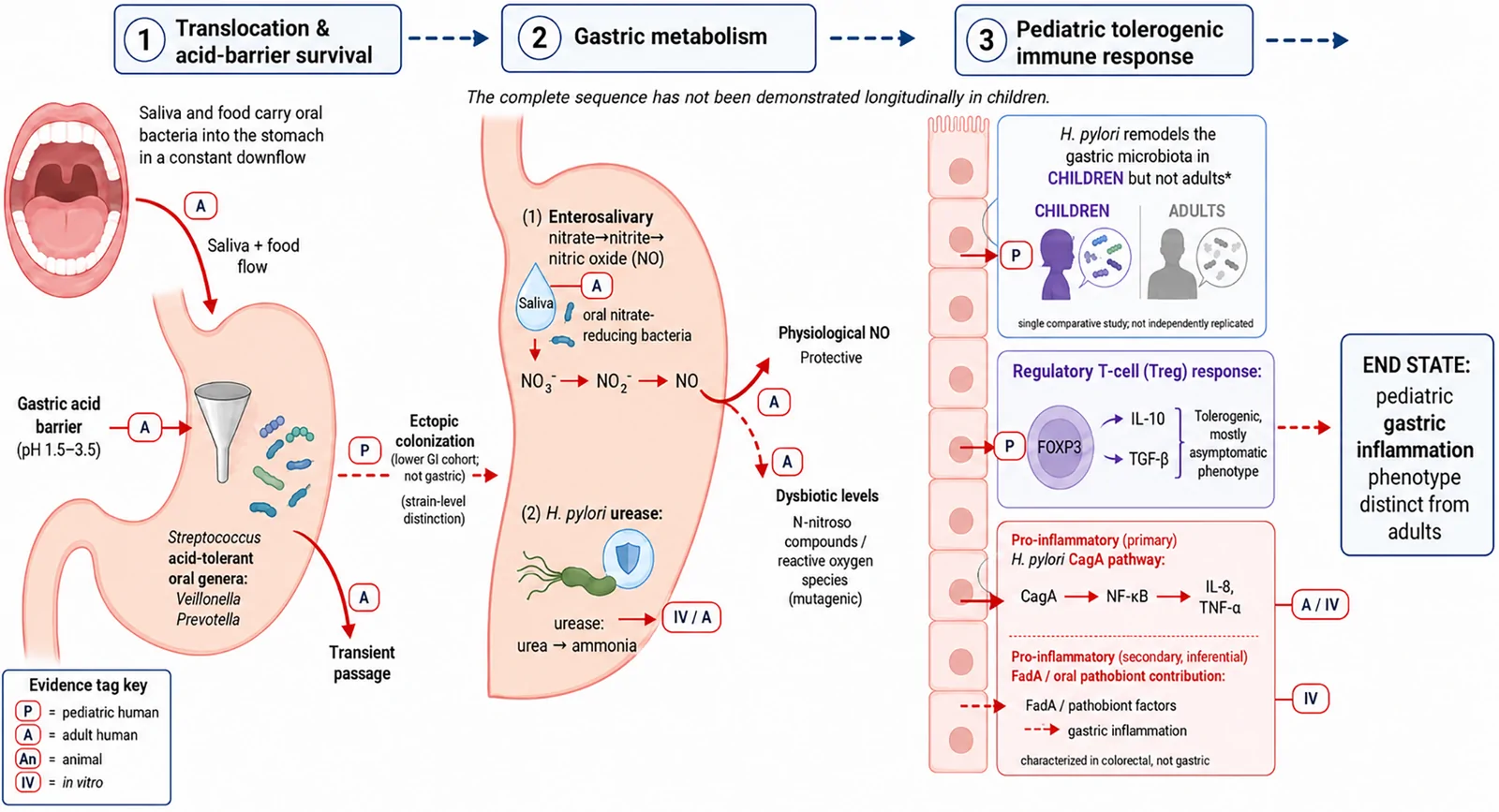
A proposed mechanistic model linking the oral-gastric axis to pediatric gastric inflammation. The model is read left to right and is a proposal for testing, not a demonstrated causal sequence: it is assembled from pediatric human, adult human, animal, gastric-cancer, and *in-vitro* evidence, and the complete sequence has not been demonstrated longitudinally in children. Stage 1 (translocation and acid-barrier survival): saliva and food carry oral bacteria into the stomach in a constant downflow; the gastric acid barrier (pH 1.5–3.5) eliminates most cells, but acid-tolerant oral genera (*Streptococcus*, *Veillonella*, *Prevotella*) survive, with outcomes ranging from transient passage to ectopic colonization. Stage 2 (gastric metabolism): the enterosalivary nitrate–nitrite–nitric oxide pathway and *H. pylori* urease generate output that is double-edged—physiological nitric oxide is protective, whereas dysbiotic levels yield N-nitroso compounds and reactive oxygen species. Stage 3 (pediatric tolerogenic immune response): in a single comparative study that has not been independently replicated, *H. pylori* infection was associated with remodeling of the gastric microbiota in children but not in adults, accompanied by an expanded regulatory T-cell response (FOXP3, IL-10, TGF-β) that is tolerogenic and mostly asymptomatic, while a shared pro-inflammatory axis operating through the CagA protein is comparatively secondary in children; a further inferential contribution from the *Fusobacterium nucleatum* adhesin FadA is shown, characterized in colorectal rather than gastric carcinogenesis. Solid arrows denote links demonstrated directly in the population identified by the accompanying tag; dashed arrows denote links inferred by extrapolation across population, species, or disease stage, and every arrow between the three stages is dashed because no cross-level transition has been demonstrated. Tags identify the evidence type for each link: P, pediatric human; A, adult human; An, animal; IV, *in vitro*; where two tags are given, the link rests on both. Animal evidence contributes to the model but is not the sole basis of any single link, so An is defined in the figure key without appearing on an arrow. Taxa and gene symbols are italicized; protein products such as CagA, FadA and FOXP3 are set in roman. NO, nitric oxide; TGF-β, transforming growth factor β; TNF-α, tumor necrosis factor α.

### 4.1 Translocation, acid-barrier survival, and ectopic colonization

The stomach is continuously inoculated from above. Saliva and food carry oral bacteria into the gastric lumen in a constant flow, and although gastric acidity and antimicrobial peptides reduce the number of viable cells by several orders of magnitude, a residue of oral-origin bacteria reliably survives the passage (Bik et al., 2006; Maldonado-Contreras et al., 2011). Two distinct outcomes follow: bacteria may translocate continuously and remain transient passengers, or they may locally adapt and establish long-term residency—a transit-versus-residency distinction documented at the strain level in children, albeit in the lower gastrointestinal tract rather than the stomach (Yersin et al., 2026). The functional consequence is that detecting an oral taxon in the stomach does not by itself prove colonization; the review must therefore distinguish transit from residency wherever the data allow.

What survives is not random. The gastric environment, at pH 1.5–3.5, selects strongly for acid-tolerant taxa, and the genera that consistently appear in the stomach—chiefly *Streptococcus*, *Veillonella*, and *Prevotella*—are precisely the acid-resistant members of the oral community (Bik et al., 2006; Maldonado-Contreras et al., 2011). This selective filter explains why the prevalent gastric genera (*Streptococcus*, *Prevotella*, *Fusobacterium*, *Veillonella*, *Neisseria*, *Haemophilus*) are overwhelmingly oral-associated (Zhang et al., 2023), and it provides the mechanistic basis for the taxonomic overlap that **Table 2** exploits. *H. pylori* itself solves the acid problem differently, neutralizing its local microenvironment through urease-generated ammonium rather than through intrinsic acid tolerance (Yang et al., 2025)—a point that matters because it makes *H. pylori* colonization partly independent of the acid filter that governs the other oral taxa: urease, together with flagellar motility and outer-membrane adhesins, is what allows the organism to traverse the mucus layer, buffer its microenvironment, and attach to the epithelium in the first place (Cheok et al., 2021). The acid-buffering trick is not unique to it: other urease-positive species—including gastric isolates of *Staphylococcus epidermidis* and the oral streptococcus *Streptococcus salivarius*—can colonize the stomach and themselves modulate *H. pylori*-associated gastric pathology and host immune responses (Shen et al., 2022).

Beyond mere survival, specific oral species establish in the stomach and acquire a pathogenic role. Once colonizing the gastric mucosa, the oral-associated species *Streptococcus anginosus* and *Prevotella melaninogenica* have been associated with gastric inflammation and carcinogenesis (Lei et al., 2024). The route by which such oral taxa reach and hold the stomach is itself mechanistically grounded: keystone oral organisms including *Fusobacterium nucleatum* and *Porphyromonas gingivalis* coaggregate directly with *H. pylori in vitro*—an interaction blocked by lysine or arginine—plausibly ferrying it from mouth to mucosa (Andersen et al., 1998); this transport role is inferred from *in-vitro* coaggregation and has not been directly verified in the pediatric gastric mucosa; consistent with a capacity for oral residence, *H. pylori* adheres to oral keratinocytes and coaggregates with resident oral bacteria *in vitro* (Juntarachot et al., 2023). Biofilm formation may then provide the physical shelter that lets these communities persist where planktonic cells would be cleared (Percival and Suleman, 2014), and oral commensals actively abet this—exopolysaccharides from Streptococcus mutans biofilms enhance *H. pylori* oral colonization and drug tolerance, helping explain how an organism poorly suited to the mouth nonetheless persists there (Hao et al., 2026). This is the clearest demonstration that ectopic colonization is not ecologically inert: oral taxa that reach the stomach can actively contribute to mucosal pathology rather than passively marking it.

### 4.2 The metabolic axis: urease, the nitrate-nitrite-nitric oxide pathway, and microbial metabolites

The second link is metabolic. The best-characterized example is the enterosalivary nitrate-nitrite-nitric oxide (NO) pathway. Humans cannot reduce nitrate, so dietary nitrate concentrated in saliva is reduced to nitrite by oral nitrate-reducing bacteria; when this nitrite-rich saliva reaches the acidic stomach, it is protonated and yields NO and a spectrum of reactive nitrogen species (Yang et al., 2025). The bacterial agents of the first step are identifiable: metagenomic analysis of tongue-surface communities resolved multiple nitrate-reducing species, several of which were previously unrecognized in this role (Hyde et al., 2014). The pathway is therefore mechanistically explicit and diet-dependent, and it links specific, salivary-accessible taxa (*Rothia*, *Neisseria*, *Veillonella*) to a chemically reactive output delivered directly to the gastric mucosa. This redox dialog is increasingly read as a two-way communication channel between the oral and gut microbiota (Paiva et al., 2024), and its capacity is modifiable: the nitrate-reducing function of the oral community is measurably impaired in periodontitis, tying oral-health status to the nitrite—and ultimately nitric oxide—supply reaching the stomach (Rosier et al., 2024).

This output is double-edged, and the distinction is central to interpreting any metabolic signature. At physiological levels, gastric NO supports mucosal blood flow and has antimicrobial effects. At dysbiotic levels, the same chemistry turns harmful: lactic acid bacteria that reduce nitrate to nitrite drive the formation of N-nitroso compounds and reactive oxygen species, with mutagenic consequences (Mowat et al., 2000; Viani et al., 2000), and their expansion is accompanied by overgrowth of oncobacteria such as *Veillonella*, *Prevotella*, *Fusobacterium*, and *Leptotrichia* (Castaño-Rodríguez et al., 2017; Chattopadhyay et al., 2023). The oral-gastric axis review of this chemistry adds lactate and acetaldehyde to nitrite as metabolites that promote malignant transformation (Salaspuro, 2011; Maejima et al., 2015); sustained co-exposure to salivary nitrite and acetaldehyde has been proposed to act in combination in the upper gastrointestinal tract, plausibly amplifying endogenous N-nitroso compound formation (Eisenbrand et al., 2022). For a pediatric gastritis review, the relevance is upstream of cancer—the same metabolites that are carcinogenic over decades are pro-inflammatory in the short term—but the continuity of the pathway is what makes early-life oral dysbiosis plausibly consequential; this metabolic chain has been characterized largely in adult and mechanistic settings, and its quantitative applicability to the pediatric gastric mucosa is inferential rather than directly demonstrated. Consistent with a readable oral footprint of gastric change, integrated metabolome-microbiome profiling of the tongue coating distinguishes patients with gastric precancerous lesions from controls (Hao et al., 2021).

### 4.3 Mucosal immunity and the divergence of pediatric from adult inflammation

The third and most pediatric-specific link is immunological, and it is where the verified primary literature is strongest. The pivotal observation, which comes from a single comparative study and has not been independently replicated, is that *H. pylori* infection was associated with remodeling of the gastric microbiota in children but not in adults: in a cohort of 86 children and adults, the gastric communities of uninfected individuals were similar across age, yet *H. pylori*-infected children—and not infected adults—showed significant shifts in multiple high-abundance taxa, more diverse microbiota, depleted *Firmicutes*, and expanded non-*Helicobacter Proteobacteria* relative to infected adults (Brawner et al., 2017), the pediatric half of which is independently corroborated by pediatric gastric-microbiota cohorts reporting reduced diversity and distinct community structure in infected children (Llorca et al., 2017; Zheng et al., 2021, Zheng et al., 2022); these pediatric cohorts lack a matched adult comparison arm, however, and so corroborate the remodeling in children rather than the children-but-not-adults contrast itself, which to date rests on this single direct comparison. On this evidence the pediatric stomach appears to respond to *H. pylori* as a microbial-ecological event in a way the adult stomach does not. That single comparison is the empirical core of this review’s argument that adult signatures cannot be extrapolated to children, and its independent replication is correspondingly the most consequential outstanding test in the field.

The immune correlate of that ecological shift explains why children are clinically different. Children with restructured gastric microbiota expressed higher levels of FOXP3, IL-10, and TGF-β, consistent with an expanded regulatory T-cell response (Brawner et al., 2017) (an immune sub-analysis of 11 infected children); independently, pediatric *H. pylori* gastritis has been characterized by precisely this regulatory T-cell signature (Harris et al., 2008). Head-to-head comparisons of infected children and adults make the contrast explicit: children mount a more tolerogenic response built on regulatory T cells and immunosuppressive cytokines such as IL-10 (Reyes, 2022), and the gastric Th17/Treg balance—indexed by FOXP3 and IL-17A expression—differs between the two age groups (Yörgüç et al., 2021); pediatric gastric mucosa also shows a distinctive CD8+ T-lymphocyte involvement in this setting (Yang et al., 2024). The interpretation is that pediatric mucosa mounts a more tolerogenic response to *H. pylori*, which dampens overt gastric disease and plausibly underlies the long-standing clinical observation that children tolerate *H. pylori* better than adults do. This reframes one of the field’s central puzzles—why pediatric infections so often remain asymptomatic—as an immunological feature rather than an anomaly —and cautions directly against importing adult inflammatory readouts into pediatric diagnostics.

Against this tolerogenic background, the inflammatory pathways that do operate are shared in kind if not in degree. Both *H. pylori* and ectopically colonizing oral pathobionts engage mucosal pro-inflammatory signaling—the IL-8 and TNF-α axes prominent in the adult gastritis literature (Crabtree et al., 1994). The molecular drivers are well defined: *H. pylori* CagA, delivered by a type IV secretion system, lowers IκB and sensitizes epithelial cells to NF-κB activation, mutually reinforcing inflammation and carcinogenesis (Suzuki et al., 2015), and CagA-positive strains directly elevate epithelial IL-8 secretion (Fazeli et al., 2016); among oral pathobionts, the *F. nucleatum* adhesin FadA binds E-cadherin to activate β-catenin and pro-inflammatory signaling, a mechanism characterized in colorectal carcinogenesis whose relevance to the gastric mucosa is inferential and has not been directly verified there (Rubinstein et al., 2013). It is the balance between this pro-inflammatory drive and the pediatric regulatory response that determines phenotype. This balance is also where *H. pylori*-positive and non-*H. pylori* gastritis are likely to diverge: in the absence of *H. pylori*, the diversifying community of oral-associated taxa (*Campylobacter*, *Neisseria*, *Prevotella*, *Streptococcus*, *Veillonella*) (Hsieh et al., 2018) supplies an alternative inflammatory stimulus, and the differing salivary signatures reported for *H. pylori*-negative gastritis—notably the low *F. nucleatum* and *P. gingivalis* levels distinctive of that state (Contaldo et al., 2021)—may be downstream reporters of which inflammatory route is active.

## 5 Salivary and oral microbial signatures as non-invasive biomarkers

The clinical case for a non-invasive readout of pediatric gastric status rests on a structural argument developed in the preceding sections: the stomach is colonized in large part by acid-tolerant taxa of oral origin, and the saliva that seeds this community is itself trivially accessible. If the microbial composition of the stomach is partly mirrored in and continuously replenished by the oral cavity, then salivary or oral microbial features could, in principle, stratify children without endoscopy. This section evaluates that proposition across four candidate signature classes—taxonomic, *H. pylori*-specific molecular, metabolic/volatile, and the analytical platforms that read them—and is explicit throughout about where the supporting evidence is pediatric and where it is extrapolated from adults. The proposed mapping of these signature classes onto *H. pylori*-positive versus non-*H. pylori* pediatric gastritis, together with the evidence maturity of each candidate, is shown in **Figure 2**.

### 5.1 Taxonomic signatures and differentiation of *H. pylori*-positive versus negative gastritis

A recurring set of genera dominates the diseased gastric mucosa across studies, and almost all of them are oral-associated. A meta-analysis of mucosal microbiota in gastric carcinogenesis identified *Fusobacterium*, *Parvimonas*, *Veillonella*, *Prevotella*, and *Peptostreptococcus* as consistently enriched in gastric carcinogenesis compared with superficial gastritis (Liu et al., 2022). Independent surveys of the gastric community converge on *Streptococcus*, *Prevotella*, *Fusobacterium*, *Veillonella*, *Neisseria*, and *Haemophilus* as the most prevalent genera (Zhang et al., 2023), and the genera most reproducibly detected across half a century of gastric studies are the same oral-associated set (Bik et al., 2006; Maldonado-Contreras et al., 2011). The overlap is not incidental: only acid-resistant oral taxa—chiefly *Streptococcus*, *Veillonella*, and *Prevotella*—survive transit through gastric acid to establish in the stomach (Maldonado-Contreras et al., 2011). Recent syntheses of the oral-microbiota-gastric-cancer literature stress that each oral niche—tongue coating, saliva, dental plaque—harbors a distinct candidate-marker set, while cautioning that between-study heterogeneity remains a barrier to consensus on a signature (Xia et al., 2024).

Translating this into a differential signature is less straightforward because the direction of change depends on context. In *H. pylori*-negative gastritis, the diversifying community is populated by *Campylobacter*, *Neisseria*, *Prevotella*, *Streptococcus*, and *Veillonella* (Hsieh et al., 2018), whereas an exploratory salivary study found that low levels of *F. nucleatum* and *P. gingivalis* distinguished *H. pylori*-negative chronic gastritis without cancerization (Contaldo et al., 2021). Niche matters as much as taxon: tongue-coating signatures appear comparatively stable across lifestyle confounders (Xu et al., 2021) and have been proposed as gastric-disease screening tools (Sun et al., 2018), while a large prospective study across Asian, African American, and European American cohorts linked specific oral taxa to subsequent gastric-cancer risk (Yang et al., 2022). The same taxon can therefore move in opposite directions depending on disease stage and specimen, which cautions against any single-taxon classifier. Even so, the salivary community carries disease information at the level of the whole profile: the saliva microbiome is measurably altered in patients with gastritis (Chen et al., 2022a), and a population-based study found oral microbiome dysbiosis—across saliva, subgingival, and buccal niches—associated with precancerous upper-gastrointestinal lesions, with specific oral sites flagged as the most promising biomarker sources (Sadeghi et al., 2024). A caution must be stated explicitly here, because it recurs throughout this section: the candidates above are drawn from a heterogeneous set of target conditions—gastric cancer, precancerous lesions, and *H. pylori*-positive or -negative gastritis—that are not interchangeable endpoints. Evidence generated in gastric-cancer cohorts (the carcinogenesis meta-analysis and the prospective gastric-cancer-risk study cited above) cannot be assumed to transfer to pediatric gastritis, and **Table 3** accordingly grades such gastric-cancer-derived signatures against the target condition for which they were actually generated, and treats their transfer to pediatric gastritis as an untested extrapolation. Candidate taxa, their reported direction, the population in which they were observed, and the specimens are consolidated in **Table 2**.

The pediatric exception worth foregrounding is *Porphyromonas*. In a direct comparison of oral microbiomes in children and adults with or without oral *H. pylori*, *Porphyromonas* was the only genus significantly enriched in oral *H. pylori*-positive status in both age groups, and beta-diversity differed significantly between *H. pylori*-positive and -negative children (Ogaya et al., 2024). This is among the very few signatures with primary pediatric support rather than adult extrapolation, although that support rests on a single pediatric-adult comparison of modest size (21 children, saliva only), reports community differences rather than diagnostic accuracy, and awaits replication. It is therefore graded as pediatric evidence at Phase 1. No head-to-head comparison of candidate taxa in a common pediatric population has been performed, so no candidate is ranked against another in this review.

### 5.2 *H. pylori*-specific oral molecular markers

Where community profiling asks what is present, targeted molecular detection asks specifically whether *H. pylori* and its virulence determinants are present in the mouth. The most directly relevant pediatric dataset comes from a cross-sectional study of 100 children aged 4–18 undergoing endoscopy for gastrointestinal symptoms, in which salivary *H. pylori* was detected by PCR targeting the 23S rRNA gene and gastric infection was ascertained on Giemsa-stained biopsies: 61 children were biopsy-positive, 53 were saliva-positive, and every saliva-positive child was also biopsy-positive (Samani et al., 2026). The complete two-by-two classification is therefore recoverable—53 true-positive, 0 false-positive, 8 false-negative, and 39 true-negative results—from which we calculate a sensitivity of 86.9% (53/61; 95% CI 75.8–94.2) and a specificity of 100% (39/39; 95% CI 91.0–100), with a positive predictive value of 100% (95% CI 93.3–100) and a negative predictive value of 83.0% (95% CI 69.2–92.4). These estimates were derived by us from the counts reported in the primary study, which did not present them. Among the virulence genes examined, *cagA* was detected in eight saliva samples and *sabA* in none (Samani et al., 2026). Three qualifications constrain what this result can support. First, it is a single small cross-sectional study from one center; the confidence intervals are correspondingly wide, and the point estimates require independent replication before they can anchor a diagnostic claim. Second, the reference standard was Giemsa histology alone rather than the concordance of two or more tests that pediatric guidelines require, so misclassification of gastric status cannot be excluded and the specificity of 100% should be read against that limitation. Third, and most consequentially for interpretation, the absence of any saliva-positive, biopsy-negative case is equally consistent with two quite different explanations: an independent oral population that faithfully accompanies gastric infection, or oral contamination derived from the stomach. High specificity is precisely what reflux-derived seeding would predict, so this dataset does not discriminate between the reservoir and the contamination hypotheses. It should also be noted that the target condition here is gastric *H. pylori* infection, not histological gastritis and not disease activity. The modest virulence-gene yield likewise tempers expectations that oral virulence genotyping will, by itself, carry diagnostic weight in children. Detection chemistry itself is maturing: multiplex PCR can co-detect the *H. pylori* 16S rRNA and *cagA* genes directly from saliva (Wang et al., 2021), and salivary point-of-care biosensors—paired monoclonal-antibody lateral-flow and electrochemical platforms—now report rapid, quantitative *H. pylori* detection without endoscopy or laboratory processing (Zan et al., 2026).

A second and considerably larger pediatric dataset addresses the same target condition. In 500 children undergoing elective gastroscopy for gastrointestinal symptoms at two centers, salivary exosomal DNA was assayed by quantitative PCR for the *H. pylori* 23S rRNA gene, and infection was defined by concordant histopathology and rapid urease testing, with discordant pairs adjudicated by urea breath or stool antigen testing (Song et al., 2026). Of the 500 children, 158 (31.6%) were infected. The assay yielded 148 true-positive, 68 false-positive, 10 false-negative, and 274 true-negative results, giving a sensitivity of 93.7% (148/158; 95% CI 88.7–96.9) and a specificity of 80.1% (274/342; 95% CI 75.5–84.2), with a positive predictive value of 68.5% (95% CI 61.9–74.7) and a negative predictive value of 96.5% (95% CI 93.6–98.3); the confidence intervals were calculated by us from the reported counts. Among 113 infected children, salivary exosomal and gastric-tissue genotyping agreed on clarithromycin resistance in 103 of 113 (91.2%, κ = 0.72).

Two features of this cohort matter more than its headline sensitivity. Its reference standard is the concordance of two gastric tests, which is methodologically stronger than the Giemsa histology used alone in the smaller cohort above, so its estimates are less exposed to misclassification of gastric status, although it is not identical to the biopsy-based algorithm specified in current pediatric guidelines and discordant pairs were adjudicated by non-invasive testing. Its specificity of 80.1% points in the opposite direction from that cohort’s 100%: 68 of 342 children without gastric infection were salivary-positive. Reflux from a stomach correctly classified as uninfected cannot produce that result, so it requires another explanation — an oral population not tracking gastric colonization, assay cross-reactivity, amplification of DNA from non-viable organisms, or misclassification by the reference standard. The two pediatric datasets therefore disagree on precisely the parameter that bears on the reservoir question, and the disagreement is informative in itself: oral *H. pylori* positivity is not a simple readout of gastric status. The clinical consequence follows arithmetically. At an infection prevalence of 31.6%, a positive predictive value of 68.5% means that roughly one positive result in three is false, and the reported specificity sits below that of the stool antigen and urea breath tests against which such an assay would compete. Neither pediatric cohort has been externally validated, so the conclusion of this review is unchanged.

A methodological caveat must travel with these markers. Co-culture work shows that non-invasive *H. pylori* detection by PCR and loop-mediated isothermal amplification (LAMP) is prone to false positives, in part through cross-reaction with *Campylobacter* species and contamination of the oral cavity via gastroesophageal reflux (Scholz et al., 2025); even within PCR, diagnostic reliability hinges on primer design and on the threshold abundance of *H. pylori* in the specimen, which differs markedly between saliva, stool, and biopsy (Šeligová et al., 2020). Positive oral *H. pylori* results, therefore, require careful interpretation, and *vacA*-based oral detection—frequently cited in the adult literature—should not be advanced as a pediatric marker without a verified primary pediatric source.

### 5.3 Metabolic and volatile signatures

Beyond taxonomy, the functional output of the oral-gastric community offers a complementary readout. The enterosalivary nitrate-nitrite-nitric oxide pathway is the best-characterized example: dietary nitrate is concentrated in saliva and reduced to nitrite by oral nitrate-reducing bacteria, which, on reaching the acidic stomach, generate nitric oxide and reactive nitrogen species (Hyde et al., 2014; Yang et al., 2025). The bacterial communities responsible are identifiable—metagenomic analysis of tongue surfaces resolved multiple nitrate-reducing species, several not previously recognized in this role (Hyde et al., 2014). In adults, this functional layer is already being read diagnostically: salivary metabolomic profiles discriminate gastric cancer from controls and shift after gastrectomy (Dong et al., 2024), the salivary metabolome is likewise altered in chronic erosive gastritis—a pre-malignant state closer to the pediatric disease spectrum (Liu et al., 2023)—and volatile read-outs are advancing, with mid-infrared exhaled-breath sensing proposed for rapid *H. pylori* detection (Flores Rangel et al., 2025). Because this output is diet-dependent and tied to specific taxa (*Rothia*, *Neisseria*, *Veillonella*), metabolite- and volatile-based signatures are mechanistically grounded but currently lack pediatric gastritis-specific validation. On the framework of Section 2 they rest on adult and non-human evidence at Phase 1, and they are graded accordingly in **Table 3** and **Figure 2**; the word emerging is avoided here because it was previously used in a sense that conflicted with that grading.

### 5.4 Analytical and computational platforms

The signatures above are only as useful as the platforms that detect them. 16S rRNA amplicon sequencing underpins most of the taxonomic evidence cited here and resolves communities to the genus level, which is adequate for the *Porphyromonas* and broad oral-associated genus signals but insufficient for species- or strain-level discrimination (Johnson et al., 2019). Shotgun metagenomics adds functional and strain-level resolution and has been used to track strain-level translocation of oral bacteria into the lower gastrointestinal tract in children, albeit in a stunting cohort rather than a gastritis cohort (Yersin et al., 2026). Targeted quantitative PCR and droplet digital PCR offer the quantification and point-of-care friendliness needed for a deployable test (Doyle et al., 2025); quantitative PCR, not the droplet-digital format, confirmed the *Campylobacter concisus* tongue-coating signature in paired tongue-coating and gastric-fluid specimens, and only in an adult cohort (Cui et al., 2019). Layered onto these, machine-learning classifiers can integrate multi-feature panels: in adults, models built on salivary genera, tongue-coating microbial proteins, and even tongue images have reached areas under the curve near 0.9 in multicenter cohorts (Yuan et al., 2023; Chen et al., 2024; Kim et al., 2026). The approach continues to scale: a tongue-coating microbiota classifier trained on nearly 1200 participants discriminated digestive-system tumors using standard machine-learning algorithms (Ma et al., 2025), and a saliva-derived transcriptomic signature, assembled partly from public datasets, has been used to build machine-learning models for gastric-cancer detection (Lopes et al., 2025); methodological reviews now catalog how such classifiers should be built and validated to characterize cancer from microbiome data (Teixeira et al., 2024). All of these classifiers were developed in adults. The exploratory salivary model most often cited in this context was itself built in an adult cohort of 293 patients spanning superficial gastritis, atrophic gastritis, and gastric cancer (Huang et al., 2021). We identified no machine-learning classifier for an oral or salivary signature that was developed or validated in children, and the small sample sizes typical of pediatric oral-microbiome studies would in any case create a real risk of overfitting, so any classifier reported on tens of children should be treated as hypothesis-generating until validated in an independent cohort.

A limitation cuts across all four signature classes and is easy to overlook: almost every candidate evaluated above is, at bottom, a detector of presence—whether *H. pylori* is present, or whether the oral or gastric community is dysbiotic—rather than a measure of disease activity. Yet the clinical task defined at the outset is not to detect the organism but to identify the minority of children in whom infection is driving mucosal disease. Because pediatric infections are often tolerogenic and frequently subclinical (Section 6.3), the presence and disease activity can dissociate, and a signature that faithfully reports colonization may still fail to report pathology. Of the candidates graded here, only those tied to the host inflammatory phenotype—the regulatory host-response signature—even attempt the harder, activity-level question; the taxonomic and *H. pylori*-specific markers should not be over-interpreted as disease-tracking on the strength of presence data alone.

Taken together, the biomarker landscape is asymmetric: the mechanistic and taxonomic rationales are strong and increasingly convergent; a small number of signatures (*Porphyromonas*; salivary 23S rRNA/*cagA*) have genuine pediatric support, but most candidate taxa and all metabolic signatures remain adult-derived. Section 6 addresses the confounders and evidence grading that determine how far these candidates can responsibly be pushed toward pediatric practice.

## 6 Translation to pediatric practice: confounders, controversies, and evidence maturity

The preceding sections establish that an oral-gastric biomarker is mechanistically plausible and that candidate signatures exist. This section asks the harder translational questions: where such a test could realistically fit in pediatric care, what pediatric-specific confounders would degrade it, and—most importantly—how mature the evidence for each candidate is. The section is deliberately conservative because the gap between a reported association and a deployable pediatric test is wide and is too often elided.

### 6.1 Where a non-invasive signature could fit—and where it must not be misused

The clinical opening is defined by what current guidelines refuse to do non-invasively. The joint ESPGHAN/NASPGHAN guidelines recommend against a test-and-treat strategy in children and require a biopsy-based diagnosis, with at least 6 gastric biopsies obtained at endoscopy (Homan et al., 2024). Endoscopy is therefore the gatekeeper, and it is invasive, resource-intensive, and impractical for repeated use. A validated salivary signature could plausibly occupy three niches: pre-endoscopic triage to prioritize which symptomatic children warrant the procedure; longitudinal monitoring where serial endoscopy is untenable; and post-eradication follow-up. None of these requires the signature to replace biopsy outright—a far lower bar than full diagnostic substitution.

A specific misuse must be guarded against. Precisely because the guidelines reject test-and-treat in children, a convenient, non-invasive marker could be co-opted into the very screen-and-treat behavior the guidelines warn against, driving unnecessary antibiotic exposure in a population where most infections are asymptomatic (Homan et al., 2024). This is not hypothetical: even under current guidance, real-world pediatric practice already shows substantial variation in how non-invasive tests are used (Rajan et al., 2024), and rising H. pylori antimicrobial resistance in children makes any avoidable treatment course more costly than it once was (Riaz et al., 2025). Any translational framing of these biomarkers must explicitly state that ease of testing is not a rationale for treatment, and that the guideline logic—investigate the cause of symptoms, not merely the presence of *H. pylori*—applies regardless of how non-invasive the test becomes.

### 6.2 Pediatric-specific confounders

Several variables that are minor or controllable in adult studies are not present in children. Diet is the most direct: nitrate exposure, largely from vegetables, shifts the oral community—increasing health-associated *Neisseria* and *Rothia* while suppressing *Prevotella*, *Veillonella*, and *Streptococcus* (Vanhatalo et al., 2018)—which is consequential given that these same genera are candidate signatures and that the nitrate pathway is itself diet-dependent (Yang et al., 2025). Diet also acts directly on gastric risk and is itself patterned by region and household: high-salt and processed, nitrate-preserved foods carry a meta-analytic gastric-cancer risk and feed N-nitroso chemistry in the stomach (Wu et al., 2021). A salivary panel read without dietary context could mistake a dietary—or socioeconomic—shift for a disease signal: household crowding and low socioeconomic status independently raise infection risk (Wang et al., 2025), and in a pediatric cohort frequent dessert and fried-food intake tracked with infection while higher household income was protective (Chen et al., 2025); comparable pediatric cohorts continue to tie infection risk to dietary pattern, household conditions, and sanitation (Nguyen Le et al., 2024; Sherpa et al., 2025).

Oral hygiene and dental development add further noise. Poor oral hygiene is epidemiologically linked to greater gastric disease risk, and periodontal therapy measurably improves gastric *H. pylori* eradication (Ozturk, 2021; Baima et al., 2024), while in children, *H. pylori* carriage correlates with dental hygiene and periodontal status (Lupu et al., 2025), so oral-health status is simultaneously a confounder and a potential effect modifier. Tooth eruption progressively reshapes the oral niche across childhood, but as noted in Section 3, longitudinal data linking eruption stage to oral-gastric microbial dynamics are lacking, so this confounder is currently unquantified rather than corrected. Antibiotic exposure is the most pronounced: the guidelines themselves require waiting at least 4 weeks after antibiotics before *H. pylori* testing (Homan et al., 2024), and eradication therapy markedly alters the still-developing pediatric microbiota (Sung et al., 2020). The magnitude and time-course are now reasonably well described: across 45 studies the disruption is consistent in adults but variable—even potentially protective—in children (Albush et al., 2025); meta-analysis shows successful eradication reverses gastric dysbiosis with alpha-diversity recovering within a month (Guo et al., 2022); and in adolescents the diversity lost immediately after triple therapy returns to pretreatment levels by the eradication-judgment visit (Kakiuchi et al., 2021), and longer-term pediatric follow-up after bismuth-quadruple or concomitant therapy similarly tracks gut-microbiota recovery and residual shifts (He et al., 2024). Because the perturbation is real, even when transient (Du et al., 2024), recent antimicrobial history should be a mandatory covariate in any pediatric biomarker study. Environmental factors, such as tobacco smoke exposure, further perturb the developing oral community (Baima et al., 2024).

### 6.3 Extrapolation limits, the tolerance paradox, and evidence maturity

The central interpretive hazard is extrapolation from adults. The verified evidence is unusually direct on this point, though it rests on a single comparative study: *H. pylori* infection significantly remodels the gastric microbiota in children but not in infected adults (Brawner et al., 2017), so an adult-derived signature is not merely less precise in children but may be qualitatively wrong. The handful of pediatric studies that profile the gastric community directly—comparing *H. pylori*-positive and -negative pediatric chronic gastritis (Chen et al., 2023) and relating gastric microbiota to infection status in children (Miao et al., 2020)—reinforce that the pediatric picture must be built from pediatric data rather than borrowed. This is compounded by the tolerance paradox—children mount a more tolerogenic, regulatory T-cell-dominated mucosal response to *H. pylori* (elevated FOXP3, IL-10, TGF-β) (Harris et al., 2008; Brawner et al., 2017), which dampens overt disease. This regulatory bias is not absolute: a concurrent Th17 component is also reported in pediatric infection, with elevated mucosal IL-17A accompanying the regulatory signature (Zheng et al., 2021), so the pediatric response is better described as a shifted Treg/Th17 balance than as purely tolerogenic. The diagnostic implication is subtle but important: if pediatric infections are frequently subclinical by virtue of immune tolerance, it is unclear which biomarker tracks—colonization, inflammation, or clinically meaningful disease—and these may dissociate in children more than in adults.

These considerations motivate the explicit grading applied in **Tables 2** and **3** under the two-axis scheme defined in Section 2. Reading the axes together rather than collapsing them changes the picture in four specific ways. Two candidates in **Table 3** reach Phase 2 on pediatric evidence, the salivary *H. pylori* PCR reported by Samani and colleagues and the salivary exosomal assay reported by Song and colleagues, and both address gastric infection rather than gastritis; the larger of the two, which used a two-test reference standard, reports a specificity of 80%, so even the best-supported pediatric candidate is not a clean readout of gastric status. Oral *Porphyromonas* has pediatric evidence but only at Phase 1, because the primary study reported community differences rather than a standardized assay with accuracy estimates. Tongue-coating *Campylobacter concisus* and the salivary *Fusobacterium nucleatum* and *Porphyromonas gingivalis* observation are both adult evidence at Phase 1, because neither primary study reported diagnostic accuracy against a reference standard. Applying the rules consistently also reverses an ordering the earlier scheme had imposed: both had been grouped with the pediatric candidates as though more mature than the adult gastric-cancer genera, whereas the gastric-cancer genera in fact reach Phase 2, on a meta-analytic classifier with an area under the curve of 0.85 for gastric cancer in adults, and these two do not. No candidate on either axis reaches Phase 3 or beyond, and none has been evaluated in children against histological gastritis or disease activity as the target condition. The maturity gap is reinforced by the fact that even reference-standard *H. pylori* detection in children shows imperfect concordance across methods (Cabrera et al., 2025), underscoring the extent of methodological groundwork required before validating any microbial signature. The distribution is sobering and summarizes the field: the two pediatric clinical-assay candidates both reach Phase 2 and address gastric infection rather than gastritis or disease activity, while the remaining pediatric entries are confined to Phase 1; the mechanistically attractive metabolic signatures rest on adult and non-human evidence at Phase 1; and the widely cited gastric-cancer genus signatures, which are the most developed candidates in **Table 3** by phase, were generated against a different target condition in a different population, so their application to pediatric gastritis remains an untested extrapolation. The most mature evidence in the field is evidence for the wrong endpoint.

The practical conclusion is that these biomarkers are, at present, instruments for research stratification and hypothesis generation rather than for clinical decision-making. Stating that plainly is not a weakness of the review but its central contribution; the roadmap in Section 7 specifies what would be required to move the most promising candidates from single-cohort discovery toward independent validation.

## 7 Discussion: integrated framework and future directions

The preceding sections can be drawn together into a single coherent picture. The oral cavity and the stomach are ecologically continuous compartments connected by a constant salivary and alimentary flow; an acid-resistant subset of the oral community survives gastric transit and, in some cases, establishes ectopically; the metabolic activity of these colonizers—nitrate reduction, lactate and acetaldehyde production, and, for *H. pylori*, urease-driven pH buffering—supplies a chemically reactive and pro-inflammatory input to the gastric mucosa; and the pediatric mucosa converts that input into a characteristically tolerogenic inflammatory phenotype distinct from the adult response. Because each step originates at a salivary-accessible site, the axis is, in principle, legible from the mouth, which is the conceptual foundation for non-invasive pediatric diagnostics.

The honest summary of the evidence is one of asymmetry. The connectivity and mechanistic arguments are strong and convergent across many studies, and a small number of observations have genuine pediatric support—the enrichment of oral *Porphyromonas* in infected children (Ogaya et al., 2024), the diagnostic accuracy of salivary *H. pylori* detection against a biopsy-based reference standard in a pediatric endoscopy cohort (Samani et al., 2026) and in a larger 500-child cohort using an exosomal assay (Song et al., 2026), and the regulatory, tolerogenic immune signature of pediatric gastritis (Harris et al., 2008; Brawner et al., 2017). Against this, the specific biomarker candidates remain immature: as **Table 3** makes explicit, none has been validated in an independent pediatric cohort, most rest on adult or gastric-cancer data, and the controversy over whether the oral cavity is even a true *H. pylori* reservoir remains unresolved (Scholz et al., 2025). The two pediatric cohorts also disagree on specificity, 100% against 80%, so oral detection is not a settled readout of gastric status (Song et al., 2026). Of the candidates graded here, none currently addresses the activity-level endpoint that the clinical rationale requires: the triage, monitoring, and post-eradication uses proposed above all presuppose a marker of disease activity, whereas every graded candidate except the (still hypothetical) host-response signature developed below as hypothesis H3 detects only presence. This mismatch between the motivating clinical question and the available readouts is, at present, the principal barrier to translation—more limiting than the absence of pediatric validation per se.

### 7.1 Testable hypotheses

To move from narrative plausibility to testable science, we propose the following falsifiable hypotheses. These are framed as proposals for future work, not as demonstrated conclusions. (H1) A composite salivary signature—for example, the relative abundance of *Porphyromonas* together with a panel of oral-associated genera—stratifies *H. pylori*-positive from non-*H. pylori* pediatric gastritis with clinically useful discrimination, independent of any single *H. pylori*-specific marker (Hsieh et al., 2018; Contaldo et al., 2021; Ogaya et al., 2024). This is the most speculative of the three hypotheses and is offered as such: its pediatric grounding is thin, because the differentiation evidence is largely adult-derived. The gastric community profile it invokes comes from an adult gastric-cancer cohort (Hsieh et al., 2018), and the salivary observation from a small adult exploratory study (Contaldo et al., 2021), while the pediatric study establishes oral *H. pylori* carriage rather than differentiation of *H. pylori*-negative gastritis (Ogaya et al., 2024). (H2) Oral *H. pylori* positivity in children is predominantly an indirect reporter of gastric infection—reflecting transient seeding or reflux—rather than evidence of an independent, stably colonizing oral population; this predicts that oral *H. pylori* signal should track gastric status but lose independent predictive value once gastric status is known; the 68 salivary-positive, gastric-negative children reported in the larger pediatric cohort (Song et al., 2026) are the first pediatric data to bear against it (Scholz et al., 2025; Samani et al., 2026). (H3) The pediatric tolerogenic response leaves a microbial-ecological footprint, such that a salivary or gastric signature associated with the regulatory phenotype (FOXP3/IL-10/TGF-β-high) distinguishes asymptomatic colonization from symptomatic disease (Harris et al., 2008; Brawner et al., 2017). Each hypothesis is constructed to be refutable with achievable study designs, and each map directly onto a candidate signature graded in **Table 3**.

### 7.2 Future directions

Realizing this agenda requires coordinated progress on five fronts. First, longitudinal pediatric cohorts: the decisive deficit is the near absence of prospective pediatric data spanning the developmental windows during which the oral and gastric communities co-assemble (Ogaya et al., 2024; Lupu et al., 2025); birth cohorts with paired oral and gastric sampling are needed to establish whether early oral colonization causally shapes later gastric disease. Second, multi-omics integration with viability resolution: combining 16S profiling with shotgun metagenomics and metabolomics would link taxonomy to functional output (Hyde et al., 2014; Johnson et al., 2019), strain-level analysis could distinguish transit from residency (Yersin et al., 2026), and methods separating viable from non-viable organisms are needed to resolve the culture-negative/PCR-positive discordance that sustains the reservoir controversy (Scholz et al., 2025). Third, point-of-care assay development: targeted quantitative PCR already provides deployable quantitative detection, and was the platform used to confirm the tongue-coating *Campylobacter concisus* signature in adults (Cui et al., 2019), and adapting such assays to validated pediatric panels is a tractable step once the panels are available. Fourth, confounder-aware study standards: because diet, oral hygiene, dental-developmental stage, antibiotic exposure, and tobacco-smoke exposure all perturb the pediatric oral community (Vanhatalo et al., 2018; Ozturk, 2021; Lupu et al., 2025), studies should adopt minimum reporting standards capturing these covariates, with recent antibiotic use a mandatory covariate consistent with the four-week washout the guidelines already require (Homan et al., 2024). Fifth, standardization consortia: multi-site efforts defining standardized specimens and metrics—analogous to calls for a defined core gastric microbiota framework (Liu et al., 2019)—would allow the small, scattered pediatric datasets to be pooled and compared meaningfully, consistent with broader calls to strengthen rigor, reproducibility, and causal inference in microbiome research as analytical pipelines and artificial intelligence methods proliferate (Caminero et al., 2025).

Those five fronts describe the field; a single concrete study design would move it. The first tractable step is a prospective diagnostic-accuracy study enrolling consecutive children undergoing upper endoscopy for a clinical indication at participating centers. Oral specimens—whole unstimulated saliva, tongue coating, and supragingival plaque—would be collected before the procedure under a written protocol fixing time since the last meal, oral-hygiene practice immediately beforehand, and fasting status, then processed by a locked assay and read by staff blinded to the endoscopic and histological findings. The candidate panel, the analytic algorithm, and the decision threshold would be prespecified and registered before recruitment opens, so that the study reports a locked rule rather than the best rule discoverable after the fact. The reference standard would be biopsy-based and defined in advance: infection status by concordance of at least two of histology, culture, and rapid urease testing, and gastritis by centrally reviewed, blinded grading of gastric biopsies. Age, dentition stage, dietary pattern, oral-health status, proton-pump inhibitor use, and antibiotic exposure within the guideline-mandated washout would be recorded before the index test is read and specified as covariates in advance rather than explored afterwards. Sample size would be calculated for a prespecified lower confidence bound on sensitivity and specificity rather than for a significance test, and reporting would follow the STARD framework.

Two limitations of that design should be stated at the outset rather than discovered at the end. First, children referred for endoscopy are enriched for disease relative to the symptomatic community population in which a triage test would actually be used, so accuracy estimates from this design are subject to spectrum bias and are best treated as an upper bound; confirmation in the intended-use population is a separate and later requirement, not a formality. Second, a single-center study cannot address between-center variation in sampling technique, assay performance, or background oral microbiota, all of which differ with diet, geography, and infection prevalence. Independent multicenter external validation with a locked threshold, followed by an evaluation of whether the test actually changes management or outcomes, would therefore be required before any claim of clinical applicability. On the framework of Section 2, the design above takes a candidate from Phase 1 or 2 to Phase 4; nothing short of the final step reaches Phase 5.

### 7.3 Limitations of this review

This review has limitations of its own that bear on how its conclusions should be read. It is a narrative review supported by a structured search rather than a systematic review: it was not registered, it drew on a single database, so records indexed elsewhere but not in PubMed will have been missed, and studies published after 11 June 2026 are not captured. The concept blocks did not cover exosome-based assays, and one eligible pediatric study on that platform was retrieved only by hand-searching during revision, which shows the search to have been sensitive to the vocabulary we chose rather than exhaustive. We did not perform a formal risk-of-bias assessment with an instrument such as QUADAS-2, or any quantitative synthesis, and we therefore cannot exclude that a different team applying the same criteria would include a somewhat different set of studies.

The grading itself is a second limitation. The two axes and the four application rules set out in Section 2 are prespecified and reproducible, but the framework is one we assembled for this purpose rather than a validated grading instrument, and assignment at the boundary between Phase 1 and Phase 2 rests on a judgement about whether an assay was standardized. Different appraisers might place borderline candidates differently, and the grades in **Tables 2** and **3** should be read as a transparent statement of our reasoning rather than as a measurement.

Three limitations follow from the underlying literature rather than from our handling of it. The pediatric evidence base is small, and several candidates rest on single studies from single center, so publication bias toward positive findings cannot be excluded even though discordant studies were sought deliberately. The pediatric Phase 2 evidence rests on only two studies, neither externally validated and one of them small and single-center, so the clinical-assay evidence is more fragile than the number of graded pediatric candidates suggests. Much of the mechanistic evidence is adult, animal, or *in-vitro*, and its applicability to children is assumed rather than demonstrated—the point the model in Section 4 makes explicit rather than resolves. Finally, heterogeneity across the included studies in specimen type, sampling protocol, sequenced region, bioinformatic pipeline, and case definition limits comparability, and we have not attempted to adjust for it; this is also why the review ranks no candidate against another.

## 8 Conclusion

The oral-gastric microbial axis offers a mechanistically grounded and clinically appealing route to non-invasive assessment of gastric disease in children, a population for whom the invasiveness of endoscopy and the limitations of existing non-invasive tests create an acute unmet need. Salivary and oral microbial signatures carry more pediatric evidence than any other class, and the two salivary nucleic-acid assays are the only candidates to reach the clinical-assay stage in children, but none has been validated in an independent pediatric cohort, most of the supporting evidence remains adult-derived, and extrapolation to children must be made explicit rather than assumed, given that in the only direct comparison to date *H. pylori* infection was associated with remodeling of the gastric microbiota in children but not in adults (Brawner et al., 2017). The framework also rests on a contingency the review has been candid about: the co-development of the oral and gastric communities across childhood has not been documented longitudinally, so the premise that early oral colonization shapes later gastric disease remains, for now, unproven. The decisive obstacle is not conceptual but evidentiary: pediatric-specific, longitudinally validated, viability-resolved studies are the gateway between an attractive hypothesis and a usable test—the same evidentiary path that microbiome science more broadly must traverse to reach clinical application (Gilbert et al., 2025). Until that gateway is passed, these signatures are best understood as instruments for research stratification rather than clinical decision-making—a distinction this review regards as its central, and deliberately honest, contribution.
