# Supplementary material for: The oral-gastric microbial axis in children: salivary signatures as non-invasive biomarkers for pediatric gastritis and Helicobacter pylori infection
Source: Front Cell Infect Microbiol. 2026 Sep 22;16:1934250. doi: 10.3389/fcimb.2026.1934250 (PMC13639738; doi:10.3389/fcimb.2026.1934250)
Supplement: Supplementary file 1 [file Table1.docx]

**Supplementary Table S1. PubMed search strategy**

Database: PubMed (MEDLINE), searched to 11 June 2026. Language restriction: none. Screening and grading: two authors independently, with disagreements resolved by reference to the primary articles.

Searches were run per subsection and, within each subsection, separately for reviews and meta-analyses (set A) and for primary studies (set B). Publication-date limits were set per subsection and are given for each query. Search strings are reproduced verbatim in a fixed-width font; straight quotation marks and truncation asterisks are intentional and must be preserved when the strings are copied into PubMed.

| **Query ID** | **Subsection** | **Set** | **Publication‑date limit** | **PubMed search string** |
| --- | --- | --- | --- | --- |
| 1-A | Disease burden and prevention (Introduction) | A — reviews and meta-analyses | 2022–2026 | ("Stomach Neoplasms"[Mesh] OR "gastric cancer"[tiab]) AND ("Helicobacter pylori"[tiab] OR "H. pylori"[tiab]) AND (eradication[tiab] OR prevention[tiab] OR incidence[tiab] OR epidemiology[tiab]) AND (Review[pt] OR Meta-Analysis[pt] OR systematic[sb]) AND 2022:2026[pdat] |
| 3.1-A | Assembly of the oral and gastric microbiota in childhood | A — reviews and meta-analyses | 2021–2026 | ("oral microbiome"[tiab] OR "oral microbiota"[tiab] OR "salivary microbiome"[tiab] OR "gastric microbiome"[tiab] OR "gastric microbiota"[tiab]) AND (development[tiab] OR assembly[tiab] OR colonization[tiab] OR establishment[tiab] OR maturation[tiab] OR acquisition[tiab]) AND (child*[tiab] OR infant*[tiab] OR pediatric*[tiab] OR paediatric*[tiab] OR "Child"[Mesh] OR "Infant"[Mesh]) AND (Review[pt] OR systematic[sb] OR Meta-Analysis[pt]) AND 2021:2026[pdat] |
| 3.1-B | Assembly of the oral and gastric microbiota in childhood | B — primary studies | 2018–2026 | ("oral microbiome"[tiab] OR "oral microbiota"[tiab] OR "salivary microbiota"[tiab] OR tongue[tiab] OR "dental plaque"[tiab]) AND (longitudinal[tiab] OR cohort[tiab] OR "16S"[tiab] OR metagenom*[tiab] OR development[tiab] OR maturation[tiab]) AND (infant*[tiab] OR child*[tiab] OR neonat*[tiab] OR "Infant"[Mesh] OR "Child"[Mesh]) NOT (Review[pt] OR Meta-Analysis[pt]) AND 2018:2026[pdat] |
| 3.2-A | Oral–gastric continuity and the niches of oral H. pylori | A — reviews and meta-analyses | 2020–2026 | (("Helicobacter pylori"[Mesh] OR "Helicobacter pylori"[tiab] OR "H. pylori"[tiab]) AND (oral[tiab] OR saliva*[tiab] OR "dental plaque"[tiab] OR "oral-gut"[tiab] OR "oral-gastric"[tiab] OR oropharyn*[tiab])) AND (Review[pt] OR systematic[sb] OR Meta-Analysis[pt]) AND 2020:2026[pdat] |
| 3.2-B | Oral–gastric continuity and the niches of oral H. pylori | B — primary studies | 2015–2026 | ("Helicobacter pylori"[tiab] OR "H. pylori"[tiab]) AND (saliva*[tiab] OR "dental plaque"[tiab] OR "oral cavity"[tiab] OR tongue[tiab] OR subgingival[tiab]) AND (detect*[tiab] OR PCR[tiab] OR culture[tiab] OR prevalence[tiab] OR concordance[tiab] OR "16S"[tiab]) NOT (Review[pt] OR Meta-Analysis[pt]) AND 2015:2026[pdat] |
| 3.3-A | The reservoir question: a genuine and unresolved controversy | A — reviews and meta-analyses | 2018–2026 | ("Helicobacter pylori"[tiab] OR "H. pylori"[tiab]) AND ("dental plaque"[tiab] OR oral[tiab] OR saliva*[tiab]) AND (reservoir[tiab] OR reinfection[tiab] OR recurrence[tiab] OR persistence[tiab] OR transmission[tiab]) AND (Review[pt] OR systematic[sb] OR Meta-Analysis[pt]) AND 2018:2026[pdat] |
| 3.3-B | The reservoir question: a genuine and unresolved controversy | B — primary studies | 2010–2026 | ("Helicobacter pylori"[tiab] OR "H. pylori"[tiab]) AND ("dental plaque"[tiab] OR oral[tiab] OR saliva*[tiab]) AND (reservoir[tiab] OR reinfection[tiab] OR recurrence[tiab] OR eradication[tiab] OR "viable but non-culturable"[tiab] OR coccoid[tiab]) NOT (Review[pt] OR Meta-Analysis[pt]) AND 2010:2026[pdat] |
| 4.1-A | Translocation, acid-barrier survival, and ectopic colonization | A — reviews and meta-analyses | 2020–2026 | ("Helicobacter pylori"[tiab] OR "H. pylori"[tiab]) AND (colonization[tiab] OR "acid resistance"[tiab] OR "acid survival"[tiab] OR urease[tiab] OR biofilm[tiab] OR adhesion[tiab] OR coaggregation[tiab] OR virulence[tiab]) AND (Review[pt] OR systematic[sb] OR Meta-Analysis[pt]) AND 2020:2026[pdat] |
| 4.1-B | Translocation, acid-barrier survival, and ectopic colonization | B — primary studies | 2015–2026 | (("Helicobacter pylori"[tiab] OR "H. pylori"[tiab]) OR "oral bacteria"[tiab]) AND (coaggregation[tiab] OR "acid tolerance"[tiab] OR urease[tiab] OR biofilm[tiab] OR "gastric colonization"[tiab] OR translocation[tiab]) NOT (Review[pt] OR Meta-Analysis[pt]) AND 2015:2026[pdat] |
| 4.2-A | The metabolic axis: urease, nitrate–nitrite–NO, and microbial metabolites | A — reviews and meta-analyses | 2020–2026 | (("enterosalivary"[tiab] OR "nitrate-nitrite"[tiab] OR "nitrate reducing"[tiab] OR "nitric oxide"[tiab] OR "N-nitroso"[tiab] OR nitrosation[tiab] OR acetaldehyde[tiab]) AND (oral[tiab] OR saliva*[tiab] OR tongue[tiab] OR microbi*[tiab] OR gastric[tiab])) AND (Review[pt] OR systematic[sb] OR Meta-Analysis[pt]) AND 2020:2026[pdat] |
| 4.2-B | The metabolic axis: urease, nitrate–nitrite–NO, and microbial metabolites | B — primary studies | 2016–2026 | ("nitrate-reducing bacteria"[tiab] OR "enterosalivary"[tiab] OR "tongue coating"[tiab] OR acetaldehyde[tiab] OR "N-nitroso"[tiab]) AND (oral[tiab] OR saliva*[tiab] OR gastric[tiab] OR microbiome[tiab] OR microbiota[tiab]) NOT (Review[pt] OR Meta-Analysis[pt]) AND 2016:2026[pdat] |
| 4.3-A | Mucosal immunity and the divergence of pediatric from adult inflammation | A — reviews and meta-analyses | 2018–2026 | ("Helicobacter pylori"[tiab] OR "H. pylori"[tiab]) AND (immun*[tiab] OR "regulatory T"[tiab] OR Treg[tiab] OR FOXP3[tiab] OR "IL-10"[tiab] OR "TGF-beta"[tiab] OR tolerance[tiab] OR inflammation[tiab]) AND (child*[tiab] OR pediatric*[tiab] OR paediatric*[tiab] OR "Child"[Mesh]) AND (Review[pt] OR systematic[sb] OR Meta-Analysis[pt]) AND 2018:2026[pdat] |
| 4.3-B | Mucosal immunity and the divergence of pediatric from adult inflammation | B — primary studies | 2014–2026 | ("Helicobacter pylori"[tiab] OR "H. pylori"[tiab]) AND ("regulatory T"[tiab] OR Treg[tiab] OR FOXP3[tiab] OR "IL-10"[tiab] OR "TGF-beta"[tiab] OR gastritis[tiab] OR "gastric microbiota"[tiab]) AND (child*[tiab] OR pediatric*[tiab] OR paediatric*[tiab] OR adolescen*[tiab] OR "Child"[Mesh]) NOT (Review[pt] OR Meta-Analysis[pt]) AND 2014:2026[pdat] |
| 5.1-A | Taxonomic signatures and differentiation of H. pylori-positive vs negative gastritis | A — reviews and meta-analyses | 2020–2026 | (("oral microbiome"[tiab] OR "oral microbiota"[tiab] OR "salivary microbiome"[tiab] OR "salivary microbiota"[tiab] OR "tongue coating"[tiab]) AND (gastritis[tiab] OR "Helicobacter pylori"[tiab] OR "H. pylori"[tiab] OR gastric[tiab]) AND (biomarker*[tiab] OR signature*[tiab] OR diagnos*[tiab])) AND (Review[pt] OR systematic[sb] OR Meta-Analysis[pt]) AND 2020:2026[pdat] |
| 5.1-B | Taxonomic signatures and differentiation of H. pylori-positive vs negative gastritis | B — primary studies | 2017–2026 | ("salivary microbiota"[tiab] OR "salivary microbiome"[tiab] OR "oral microbiota"[tiab] OR "oral microbiome"[tiab] OR "tongue coating"[tiab]) AND (gastritis[tiab] OR "Helicobacter pylori"[tiab] OR "H. pylori"[tiab]) AND (Porphyromonas[tiab] OR Fusobacterium[tiab] OR "case-control"[tiab] OR "16S"[tiab] OR signature*[tiab]) NOT (Review[pt] OR Meta-Analysis[pt]) AND 2017:2026[pdat] |
| 5.2-A | H. pylori-specific oral molecular markers | A — reviews and meta-analyses | 2018–2026 | ("Helicobacter pylori"[tiab] OR "H. pylori"[tiab]) AND (saliva*[tiab] OR oral[tiab]) AND (PCR[tiab] OR "real-time PCR"[tiab] OR qPCR[tiab] OR "molecular detection"[tiab] OR "23S rRNA"[tiab] OR cagA[tiab] OR vacA[tiab] OR ureA[tiab]) AND (Review[pt] OR systematic[sb] OR Meta-Analysis[pt]) AND 2018:2026[pdat] |
| 5.2-B | H. pylori-specific oral molecular markers | B — primary studies | 2015–2026 | ("Helicobacter pylori"[tiab] OR "H. pylori"[tiab]) AND (saliva*[tiab] OR oral[tiab] OR mouthwash[tiab]) AND ("23S rRNA"[tiab] OR cagA[tiab] OR vacA[tiab] OR ureA[tiab] OR "real-time PCR"[tiab] OR qPCR[tiab] OR "droplet digital"[tiab]) NOT (Review[pt] OR Meta-Analysis[pt]) AND 2015:2026[pdat] |
| 5.3-A | Metabolic and volatile signatures | A — reviews and meta-analyses | 2019–2026 | ((metabolom*[tiab] OR "volatile organic compound*"[tiab] OR VOC[tiab] OR "breath test"[tiab] OR "breath analysis"[tiab] OR breathom*[tiab]) AND (gastric[tiab] OR "Helicobacter pylori"[tiab] OR "H. pylori"[tiab] OR saliva*[tiab] OR oral[tiab])) AND (Review[pt] OR systematic[sb] OR Meta-Analysis[pt]) AND 2019:2026[pdat] |
| 5.3-B | Metabolic and volatile signatures | B — primary studies | 2016–2026 | (metabolom*[tiab] OR "volatile organic compound*"[tiab] OR "exhaled breath"[tiab] OR breathom*[tiab]) AND (saliva*[tiab] OR oral[tiab] OR gastric[tiab]) AND ("gastric cancer"[tiab] OR gastritis[tiab] OR "Helicobacter pylori"[tiab] OR "H. pylori"[tiab]) NOT (Review[pt] OR Meta-Analysis[pt]) AND 2016:2026[pdat] |
| 5.4-A | Analytical and computational platforms | A — reviews and meta-analyses | 2020–2026 | (("machine learning"[tiab] OR "deep learning"[tiab] OR "artificial intelligence"[tiab] OR classifier*[tiab] OR metagenom*[tiab] OR metaproteom*[tiab] OR "absolute quantification"[tiab]) AND (microbiom*[tiab] OR microbiota[tiab]) AND (diagnos*[tiab] OR gastric[tiab] OR cancer[tiab])) AND (Review[pt] OR systematic[sb] OR Meta-Analysis[pt]) AND 2020:2026[pdat] |
| 5.4-B | Analytical and computational platforms | B — primary studies | 2018–2026 | ("machine learning"[tiab] OR "deep learning"[tiab] OR classifier*[tiab] OR "random forest"[tiab] OR metaproteom*[tiab] OR "tongue image"[tiab] OR "tongue coating"[tiab]) AND (microbiom*[tiab] OR microbiota[tiab] OR saliva*[tiab]) AND ("gastric cancer"[tiab] OR gastritis[tiab] OR "Helicobacter pylori"[tiab] OR diagnos*[tiab]) NOT (Review[pt] OR Meta-Analysis[pt]) AND 2018:2026[pdat] |
| 6.1-A | Where a non-invasive signature could fit — and where it must not be misused | A — reviews and meta-analyses | 2017–2026 | ("Helicobacter pylori"[tiab] OR "H. pylori"[tiab]) AND (guideline*[tiab] OR consensus[tiab] OR "test and treat"[tiab] OR screening[tiab] OR management[tiab] OR diagnosis[tiab]) AND (child*[tiab] OR pediatric*[tiab] OR paediatric*[tiab] OR "Child"[Mesh]) AND (Review[pt] OR Guideline[pt] OR "Practice Guideline"[pt] OR systematic[sb] OR Meta-Analysis[pt]) AND 2017:2026[pdat] |
| 6.1-B | Where a non-invasive signature could fit — and where it must not be misused | B — primary studies | 2016–2026 | ("Helicobacter pylori"[tiab] OR "H. pylori"[tiab]) AND (overdiagnos*[tiab] OR overtreat*[tiab] OR "antibiotic stewardship"[tiab] OR "antimicrobial resistance"[tiab] OR unnecessary[tiab] OR screening[tiab]) AND (child*[tiab] OR pediatric*[tiab] OR paediatric*[tiab]) NOT (Review[pt] OR Meta-Analysis[pt]) AND 2016:2026[pdat] |
| 6.2-A | Pediatric-specific confounders | A — reviews and meta-analyses | 2020–2026 | ("Helicobacter pylori"[tiab] OR "H. pylori"[tiab] OR "gastric microbiota"[tiab]) AND (diet*[tiab] OR socioeconomic[tiab] OR crowding[tiab] OR "social determinants"[tiab] OR hygiene[tiab] OR eradication[tiab] OR antibiotic*[tiab]) AND (child*[tiab] OR pediatric*[tiab] OR paediatric*[tiab] OR "Child"[Mesh]) AND (Review[pt] OR systematic[sb] OR Meta-Analysis[pt]) AND 2020:2026[pdat] |
| 6.2-B | Pediatric-specific confounders | B — primary studies | 2019–2026 | ("Helicobacter pylori"[tiab] OR "H. pylori"[tiab]) AND (diet*[tiab] OR socioeconomic[tiab] OR crowding[tiab] OR sanitation[tiab] OR "risk factor*"[tiab] OR eradication[tiab] OR "gut microbiota"[tiab]) AND (child*[tiab] OR pediatric*[tiab] OR paediatric*[tiab] OR adolescen*[tiab]) NOT (Review[pt] OR Meta-Analysis[pt]) AND 2019:2026[pdat] |
| 6.3-A | Extrapolation limits, the tolerance paradox, and evidence maturity | A — reviews and meta-analyses | 2019–2026 | ("Helicobacter pylori"[tiab] OR "H. pylori"[tiab]) AND ("gastric microbiota"[tiab] OR "gastric microbiome"[tiab] OR biomarker*[tiab] OR immun*[tiab]) AND (child*[tiab] OR pediatric*[tiab] OR paediatric*[tiab]) AND (difference*[tiab] OR "age-dependent"[tiab] OR "age-related"[tiab] OR versus[tiab] OR compared[tiab] OR distinct[tiab]) AND (Review[pt] OR systematic[sb] OR Meta-Analysis[pt]) AND 2019:2026[pdat] |
| 6.3-B | Extrapolation limits, the tolerance paradox, and evidence maturity | B — primary studies | 2015–2026 | ("Helicobacter pylori"[tiab] OR "H. pylori"[tiab]) AND ("gastric microbiota"[tiab] OR "gastric microbiome"[tiab]) AND (child*[tiab] OR pediatric*[tiab] OR paediatric*[tiab] OR adolescen*[tiab]) AND (cohort[tiab] OR "case-control"[tiab] OR "16S"[tiab] OR profiling[tiab] OR remodel*[tiab]) NOT (Review[pt] OR Meta-Analysis[pt]) AND 2015:2026[pdat] |
| 7-A | Translational and validation roadmap (Discussion) | A — reviews and meta-analyses | 2021–2026 | (microbiom*[tiab] OR microbiota[tiab]) AND (biomarker*[tiab] OR diagnostic*[tiab]) AND (validation[tiab] OR "clinical translation"[tiab] OR "external validation"[tiab] OR reproducibility[tiab]) AND (Review[pt] OR Meta-Analysis[pt]) AND 2021:2026[pdat] |

Notes. Reference lists of all included articles, of the current pediatric Helicobacter pylori guidelines, and of the reviews compared in Table 1 of the main article were additionally hand-searched; most sources published before 2010 were identified in that way rather than by the searches above. Per-query retrieval counts were not recorded at the time of searching and are therefore not reported. The biomarker concept block does not contain terms for exosomes or extracellular vesicles; one eligible pediatric study using that platform was identified by hand-searching during revision, so this table records the searches actually performed rather than an exhaustive strategy.
